# Exploration of Glitazone/Thiazolidinedione Derivatives: Molecular Design and Therapeutic Potential

**DOI:** 10.3390/bioengineering12101024

**Published:** 2025-09-25

**Authors:** Salahuddin, Avijit Mazumder, Mohamed Jawed Ahsan, Rajnish Kumar, Zabih Ullah, Mohammad Shahar Yar, Km Shabana

**Affiliations:** 1 Department of Pharmaceutical Chemistry, Noida Institute of Engineering and Technology (Pharmacy Institute), Plot No.19, Knowledge Park-2, Greater Noida 201306, Uttar Pradesh, India; rajnishkumarpharmacy@niet.co.in; 2 Department of Pharmacology, Noida Institute of Engineering and Technology (Pharmacy Institute), Plot No.19, Knowledge Park-2, Greater Noida 201306, Uttar Pradesh, India; avijitmazum@yahoo.com; 3 Department of Pharmaceutical Chemistry, Faculty of Pharmacy, Jahangirabad Institute of Technology, Jahangirabad Fort, Jahangirabad, Barabanki 225203, Uttar Pradesh, India; jawedpharma@gmail.com; 4 Department of Pharmaceutical Sciences, College of Dentistry and Pharmacy, Buraydah Colleges, Al-Qassim 51418, Saudi Arabia; izabih@gmail.com; 5 Department of Pharmaceutical Chemistry, School of Pharmaceutical Education and Research, Jamia Hamdard, New Delhi 110062, India; 6 Department of Pharmaceutical Chemistry, HIMT College of Pharmacy, Knowledge Park-1, Greater Noida 201310, Uttar Pradesh, India; shabanachoudhary6395@gmail.com

**Keywords:** thiazolidinedione, antidiabetic, antimicrobial, anti-proliferative, PPAR-γ

## Abstract

This review of thiazolidinedione or glitazone, which have a five-membered heterocyclic ring C_3_NS, shows their versatile properties in terms of pharmacological actions such as antimicrobial, antifungal, insecticidal, pesticidal, antidiabetic, anti-inflammatory, anti-proliferative, anti-neurotoxicity, anticonvulsant, anti-thyroidal, and anti-tubercular uses. While having a wide range of biological activities, the TZDs mainly act via binding to the peroxisome proliferator-activated receptor (PPAR) members. PPAR-γ are ligand-activated transcription factors, which are members of the nuclear hormone receptors group. Activations of PPAR-γ regulate cell proliferation and differentiation, glucose homeostasis, apoptosis, lipid metabolism, and inflammatory responses. This review explores the synthesis of a thiazolidinedione and its derivatives, focusing on their pharmacological profiles and antidiabetic activity. It highlights the benefits of synthesis, reaction profiles, and catalyst recovery, which may encourage further investigation into these scaffolds by researchers. Based on synthesized derivatives, some glimpses of the structure–activity relationships of some compounds have been compiled. All the synthesized derivatives have been reviewed concerning their standard drugs already available and concluded with the highly or moderately active synthesized derivatives of thiazolidinedione. The data for this review was collected by an extensive review of current scientific literature, including on the synthesis, biological evaluation, SAR, and patents (2015–25).

## 1. Introduction

**Figure 1:**
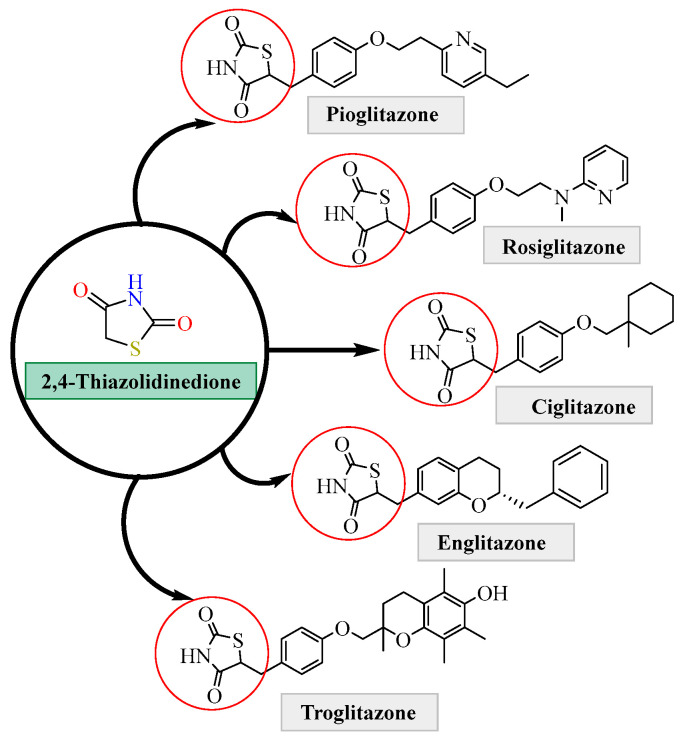
Representatives of thiazolidinedione.

Thiazolidinediones belong to the class of heterocyclic compounds with a five-membered C_3_NS ring framework which are also referred to as “glitazones” or “insulin enhancers.” Thiazolidinedione (TZD) is a word from early 1923 that describes a class of drugs used to treat type 2 diabetes (Figure 1) [1,2] which are generated from thiazolidine and additionally contain nitrogen and sulfur atoms [3,4,5] and two carbonyl groups at positions 2 and 4. In 1982, 2,4-thiazolidinediones were considered for their antidiabetic properties intensively. Ciglitazone is the first of the class, followed by Pioglitazone, Englitazone, and Troglitazone [6,7,8,9]. The first reaction for obtaining TZD was described using mono-chloroacetic acid and thiourea in an aqueous medium with cyclization [10,11].

Type 1 diabetes is an autoimmune condition resulting in the destruction of pancreatic beta-cells and absolute insulin deficiency. Type 2 diabetes is a polygenic illness marked by insulin resistance in the liver and peripheral tissue, along with relative insulin deficiency [12,13]. Insulin resistance is the precursor of type 2 diabetes, a condition in which cells in the body do not react appropriately to insulin. High blood glucose levels during pregnancy in women without a history of diabetes are known as gestational diabetes [14,15,16].

**Figure 2:**
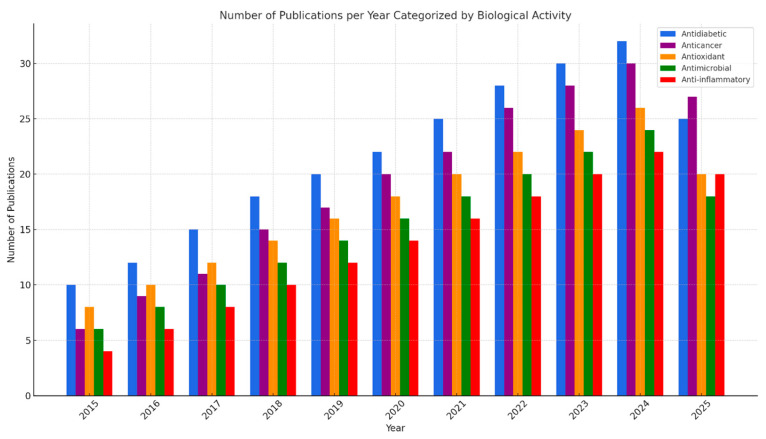
Trends in research publications on thiazolidinedione derivatives from 2015 to 2025. Data categorized by biological activity: antidiabetic, anticancer, antioxidant, antimicrobial, and anti-inflammatory.

Thiazolidinediones function as peroxisome proliferator-activated receptors gamma (PPAR-γ) agonists, which are nuclear hormones. PPAR-γ regulates the expression of many genes associated with glucose metabolism, but this represents only a small portion of its function. Upon activation, PPAR-γ associates with certain DNA sequences termed peroxisome proliferator response elements (PPREs), thus modulating the transcription of target genes. This activation enhances glucose absorption by increasing the expression of glucose transporter type 4 (GLUT4) in adipose tissue and muscle, which promote the release of adiponectin to boost insulin sensitivity and inhibits the production of pro-inflammatory cytokines such as TNF-α and IL-6. It helps to preserve pancreatic β cell structure and mass more effectively than insulin secretagogues, such as sulfonylureas. This is because TZDs reduce insulin resistance, thereby lowering the secretory burden on β cells, whereas secretagogues stimulate insulin release. Collectively, these benefits enhance glycemic regulation and diminish insulin resistance [17]. The blood glucose concentration in peripheral tissues is affected by glucose homeostasis due to the activation of PPAR-γ by thiazolidinedione; however, this predominance is primarily seen in a sedentary state. During physical exertion or energy-depleting situations, AMPK serves as the principal regulator of glucose homeostasis, surpassing the effects mediated by PPAR-γ [18,19]. By causing compensatory hyperinsulinemia, increased islet inflammation, and beta-cell lipotoxicity, insulin resistance reduces pancreatic beta-cell function [20]. Increased beta cell death and progressive reductions in insulin secretory ability are brought on by these circumstances. Rise in numerous cardiovascular risk variables are linked to insulin resistance [21]. Thiazolidinediones reduce HbA1c levels in patients with type 2 diabetes, indicating enhanced long-term glycemic regulation. HbA1c indicates the proportion of glycated hemoglobin, which correlates with average blood glucose levels over a period of 2–3 months and does not reflect tolerance to hyperglycemia or the degree of glycation. Both drugs, rosiglitazone and pioglitazone, help lower the blood glucose level as well as sensitivity of insulin, usually by reducing HbA1c levels about 0.5–1.5% [22,23,24,25,26]. Rosiglitazone reduces the long-term incidence of type 2 diabetes by slowing down, rather than completely stopping, the basic progression of the disease [26]. It raises HDL-cholesterol levels, decreases high-sensitivity C-reactive protein (hs-CRP), enhances urinary excretion of albumin and other proteins, and affects both the development and progression of diabetic nephropathy [27,28,29,30,31]. Although several TZDs have shown tolerance in specific studies, some, especially troglitazone and rosiglitazone, have been withdrawn due to significant side effects, including hepatotoxicity and increased cardiovascular risk. Other compounds, such as darglitazone and ciglitazone, have failed clinical studies because of significant toxicity [32,33,34,35]. Additional positive benefits on atherogenesis, endothelial function, ovarian steroidogenesis, and fibrinolysis have been demonstrated by research [36,37]. According to certain research, PPAR-gamma receptor stimulation can cause cancer cells to die [38]. Due to inconsistent research and other confounders, these pathways are still being studied. Thiazolidinediones have been classified as the drugs used in the management of type 2 diabetes known as glitazones. Apart from this, based on SAR or chemistry, they have been classified as TZDs with an oxymethyl linker, TZDs with an oxyethyl linker, TZDs with an oxyethylamino linker, TZD conjugates, TZDs with unusual substitutions, and TZDs with N substitution. A bibliometric chart of *thiazolidinedione* (TZD) research typically visualizes the trends in publications over time, categorized by biological activity. Based on the available literature, this graph (Figure 2) illustrates the number of research publications on thiazolidinedione derivatives from 2015 to 2025, categorized by biological activity [39].

Molecular docking of glitazones, such as pioglitazone and rosiglitazone, demonstrates significant binding to the PPARγ receptor, with binding energies of −10 to −11 kcal/mol. It establishes essential hydrogen bonds with residues such as Ser289 and Tyr473, along with hydrophobic interactions that stabilize the ligand within the receptor. However, binding interactions alone may not confirm complete agonistic activity, as inverse agonists, partial agonists, and antagonists may demonstrate similar or even superior binding affinities to PPARγ [40,41].

The main objective of the study is to evaluate the antidiabetic efficacy of TZD derivatives by examining their impact on blood glucose regulation (HbA1c levels) and insulin sensitivity. Structure–activity relationship (SAR) investigations are employed to elucidate the impact of chemical changes on biological activity. The endpoints pertain to SAR-based assessment of structural characteristics associated with biological efficacy.

### History of Thiazolidinedione

The first methodologies for the synthesis of the core thiazolidinedione structure were purportedly reported as early as 1923, and the term was accepted by IUPAC well before the 1990s. In fact, it was used by Takeda Pharmaceuticals in the late 1970s for a general drug class. Thiazolidinedione was documented to be insulin tolerant in diabetic animal models in the 1980s, and it was documented to decrease blood sugar and lipid concentrations. Following that, Isseman and Green reported the first nuclear peroxisome proliferator-triggered receptor in the 1990s. Therefore, two other isoforms of PPAR, PPAR-γ and PPAR-δ (also called PPAR-β), were known [42,43].

In another approach, ciglitazone was discovered with propitious lipid and glucose-lowering effects but profound liver toxicity [44,45]. In 1997, troglitazone (another thiazolidinedione analog) was approved by the FDA to treat type 2 diabetes, but use was discontinued due to an increased occurrence of drug-induced hepatitis. [46]. In 1999, pioglitazone and rosiglitazone were introduced. Pioglitazone was withdrawn due to the high risk of bladder cancer. The FDA revised the warning and advised that pioglitazone should not be initiated in patients with active bladder cancer and that patients with a history of bladder cancer should be treated with care [47]. Rosiglitazone was restricted in the USA and withdrawn in Europe after shreds of evidence of cardiovascular risk and death [48].

## 2. Pharmacological Importance of Thiazolidinedione as Therapeutic Agents

TZDs have reported pharmacological properties for further development as new ligands for drug discovery, including antidiabetic, anticancer, antimicrobial, antioxidant, and anti-inflammatory activities, as well as several miscellaneous effects [49].

### 2.1. Antidiabetic Activity

**Scheme 1:**
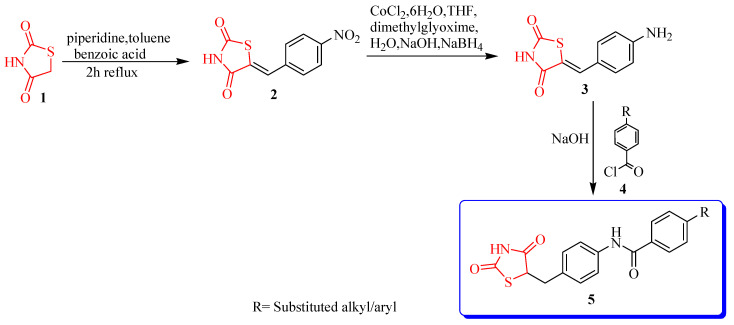
Derivatives of (2,4-dioxo-1,3-thiazolidin-5-yl)methylphenylbenzamides.

**Figure 3:**
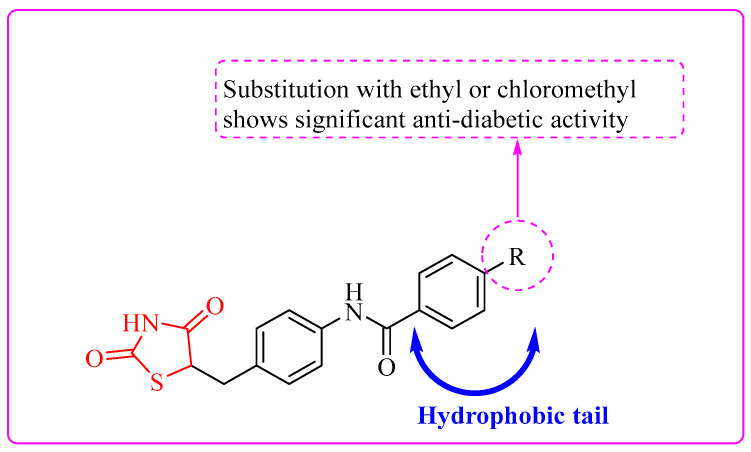
SAR of derivatives of 2,4-dioxo-1,3-thiazolidin5-yl)methylphenylbenzamides.

Several TZDs are utilized as antidiabetic agents, and their synthetic pathways are well-established. This presentation introduces novel molecules designed for development as antidiabetic agents. They are as follows:

TZD **1** was treated with 4-nitrobenzaldehyde in the presence of piperidine, toluene, and benzoic acid to give 5-(4-nitrobenzyliden)thiazolidine-2,4-dione **2**. The intermediate was then reduced to yield 5-(4-aminobenzyl)thiazolidine-2,4-dione, labeled as compound **3**. Finally, intermediate II **3** was treated with a range of substituted benzoyl chlorides **4** to afford the desired compounds (2,4-dioxo-1,3-thiazolidin-5-yl)methylphenylbenzamides **5**. The anti-hyperglycemic activity of the synthesized compounds was observed, and it was found that the compounds substituted with ethyl and chloromethyl were the most active compounds in this series compared with pioglitazone. The results indicate that the compounds exhibited notable antidiabetic effects by reducing the elevated plasma glucose associated with type 2 diabetes mellitus. Additionally, both compounds demonstrated strong binding energy in molecular docking analyses, with docking scores of −6.38 and 6.14, respectively, with standards (G-score −7.75). The detailed synthetic strategy for compound **5** was outlined in Scheme 1, with the detailed structure activity relationship (SAR) analysis shown in Figure 3. As shown in Figure 3, the phenyl R functions as a hydrophobic tail, and compounds in which R is substituted with ethyl or chloromethyl groups exhibit strong antidiabetic activity [50].

**Scheme 2:**
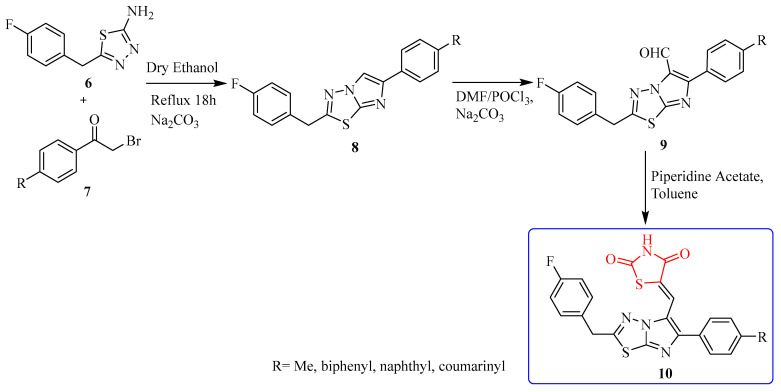
5-{[2-(4-alkyl/aryl)-6-arylimidazo[1,2][1,3,4]thiadiazol5-yl]metylene}-1,3-thiazolidine-2,4-dione.

**Figure 4:**
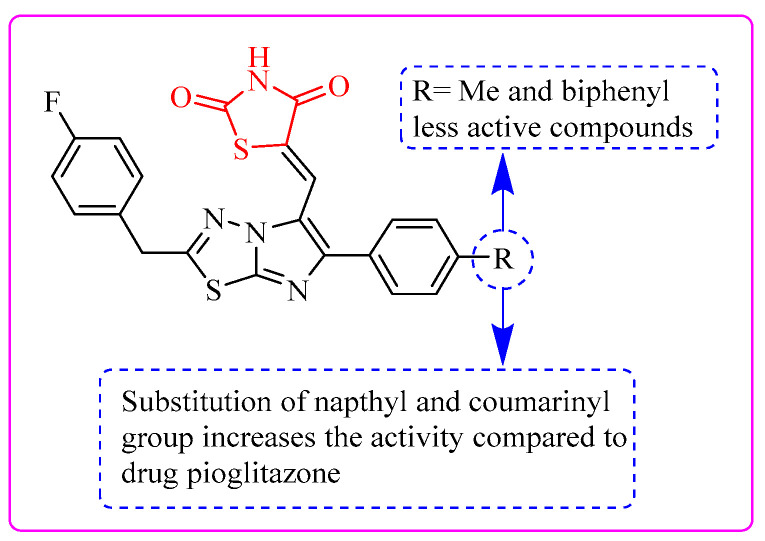
SAR of [5-{[2-(4-alkyl/aryl)-6-arylimidazo[1,2][1,3,4]thiadiazol5-yl]metylene}-1,3-thiazolidine-2,4-dione].

A series of novel 5-[2-(4-fluorobenzyl)-6-aryl-imidazo [2,1-b] [1,3,4]thiadiazol-5-ylmethylene derivatives of thiazolidine-2,4-dione **10** were produced. All compounds were produced using Knoevenagel condensation of 2-(4-fluorobenzyl)-6arylimidazo[2,1-b][1,3,4]thiadiazole-5-carbaldehydes **9** in conjunction with POCl_3_, as shown in Scheme 2. All newly synthesized compounds were evaluated for their in vivo low-glucose-level effects in male Wistar rats. Thiazolidinediones (TZDs) with naphthyl and coumarinyl substitutions had significant hypoglycemic efficacy, as demonstrated by a percentage reduction in plasma glucose levels at various dosages (10, 30, and 100 mg/kg body weight). The results indicated that these compounds exhibited a variety of hypoglycemic activities (42.48 ± 3.25 to 70.35 ± 3.14). Substituting naphthyl or coumarinyl groups into the molecule improved its biological activity, as illustrated in Figure 4. These alterations probably enhance binding affinity or pharmacokinetic characteristics [51].

**Scheme 3:**
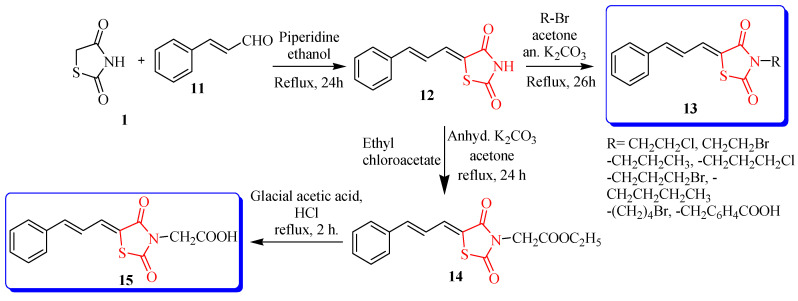
Synthesis of (Z)-3-substituted-5-((E)-3-phenylallylidene)thiazolidine-2,4-diones.

**Figure 5:**
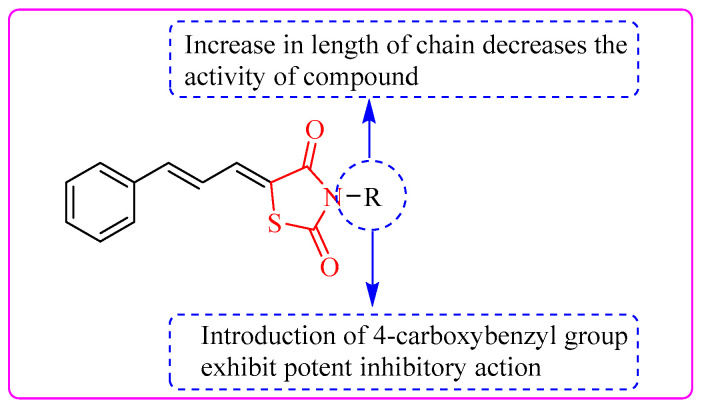
SAR of (Z)-3-substituted-5-((E)-3-phenylallylidene)thiazolidine-2,4-diones.

The reaction of commercially available TZD (**1**) with cinnamaldehyde **11** in the presence of piperidine produced (5Z)-5-[(E)-3-phenylallylidene]thiazolidine-2,4-dione **12**. Compound **12** was subsequently reacted with a number of alkyl bromides, substituted alkyl bromides, and substituted aryl bromides, using anhydrous potassium carbonate as a base in acetone. After the workup and recrystallization from methanol, the reaction yielded the pure product compound **13**, as shown in Scheme 3. The results from the in vitro assay indicate that the compound containing 4-carboxylpheny methyl demonstrates the strongest PTP-1B inhibitory activity with an IC_50_ of 6.52 mM, showing anti-hyperglycemic potential similar to that of the insulin. The SAR of 3-substituted-5-((E)-3-phenylallylidene)thiazolidine-2,4-diones derivatives is mentioned in Figure 5 [52].

**Scheme 4:**
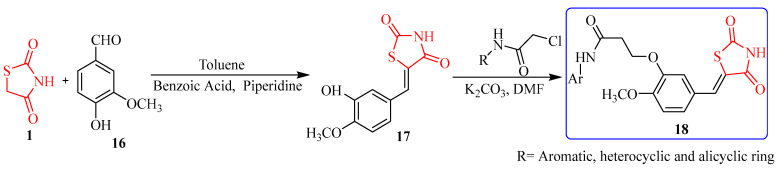
Synthetic strategy of (Z)-N-aryl-3-(5-((2,4-dioxothiazolidin-5-ylidene)methyl)-2-methoxyphenoxy)propenamide.

**Figure 6:**
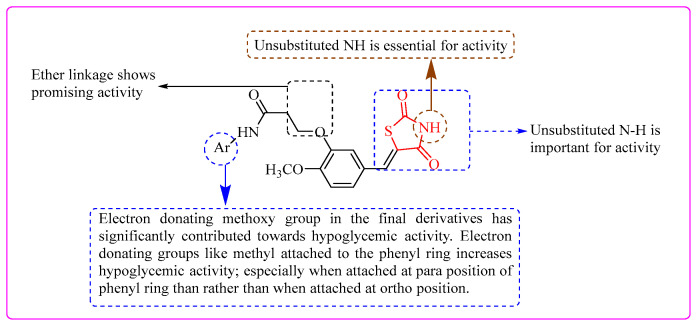
SAR of (Z)-N-aryl-3-(5-((2,4-dioxothiazolidin-5-ylidene)methyl)-2-methoxyphenoxy)propanamide.

Nikalje et al. synthesized several thiazolidinedione derivatives by reacting 4-hydroxy-3-ethoxybenzaldehyde **16** in ethanol with piperidine and benzoic acid, as outlined in Scheme 4. The alloxan-induced diabetes rat model, along with Dunnett’s *t*-test and ANOVA, was employed to evaluate the in vitro antidiabetic efficacy of each synthetic compound. From this series the synthesized compounds exhibited moderate hypoglycemic effects, with compounds **18e**, **18f**, **18b**, **18a**, and **18d** demonstrating a reduction in blood glucose levels: the SAR is illustrated in Figure 6 [53].

**Scheme 5:**
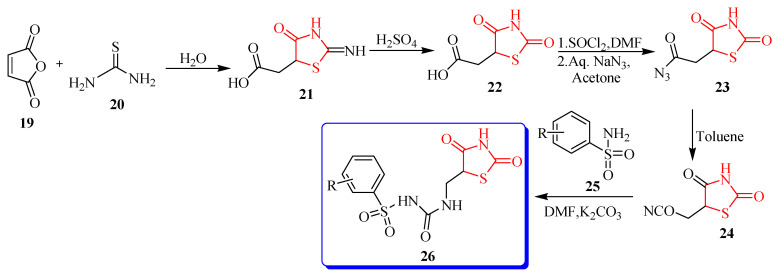
Schematic representation of 1-((2, 4-dioxothiazolidin-5-yl) methyl)-3-tosylureas.

**Figure 7:**
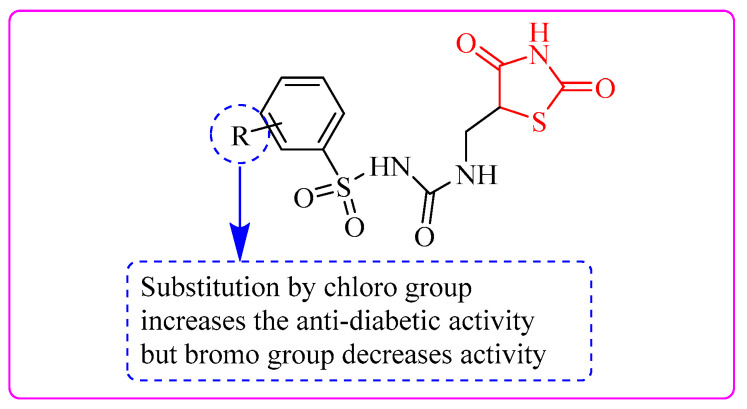
SAR of 1-((2, 4-dioxothiazolidin-5-yl) methyl)-3-tosylureas derivatives with antidiabetic activity.

Synthesis of 1-((2,4-dioxothiazolidin-5-yl) methyl)-3-tosylureas **26** by the help of benzene sulphonamides **25** has been completed starting from Furan-2,5-dione **19** and thiourea **21** in water. The intermediate was first reacted with sulfuric acid to produce 2,4-dioxo-thiazolidin-5-yl-acetic acid **(22)**. This compound was then treated with SOCl_2_ in DMF and DCM, providing a suitable synthetic route to the final target compound. The synthesized and assessed compounds show effective activity, as described in Scheme 5. These compounds substituted with H, 4-chloro, 2,4-dichloro, and 2,5dichloro significantly inhibited the rise in postprandial hyperglycemia to the tune of 15.8 (*p* < 0.01), 17.2 (*p* < 0.01), 14.3 (*p* < 0.05), and 16.5 (*p* < 0.01)%, respectively, and the SAR analysis is presented in Figure 7 [54].

**Scheme 6:**
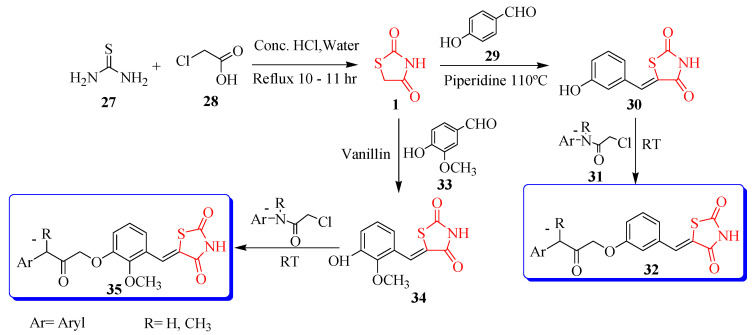
Synthetic strategy of compound **32** and **35**.

**Figure 8:**
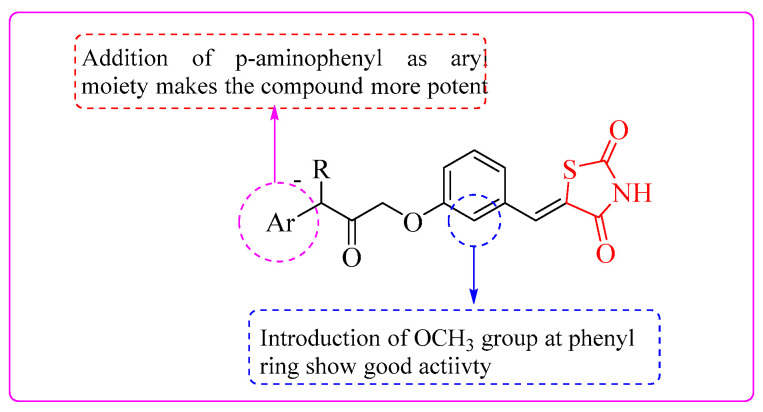
SAR of compound **32** and **35**.

In this reaction, N-(substituted)-2-{4-[(2, 4-dioxo-1, 3-thiazolidin-5-ylidene) methyl] phenoxy} acetamide **32** and [3-(2,4-Dioxo-thiazolidin-5-ylidenemethyl)-2-methoxy-phenoxy]-acetaldehydes **35** were synthesized for the determination their potential against diabetes. The reaction was initiated from thiourea **27** and chloroacetic acid **28** in concentrated hydrochloric acid and water, and was further refluxed for 10–11 h. Further, 4-Hydroxy-3-methoxy-benzaldehyde **32** was incorporated in one step and 2-Chloro-acetamide **31** in another for the end product synthesis. Synthesized compounds were evaluated on the basis of standard drugs, and those that are N-methylaniline substituted show significant activity. The synthetic scheme is mentioned in Scheme 6. The SARs of the synthesized compounds suggest that modifying the substituent group (R) on the molecule can influence its glucose-lowering capacity, with groups like nitro, chloro, or fluoro affecting related biological activities, as depicted in Figure 8 [55].

**Scheme 7:**
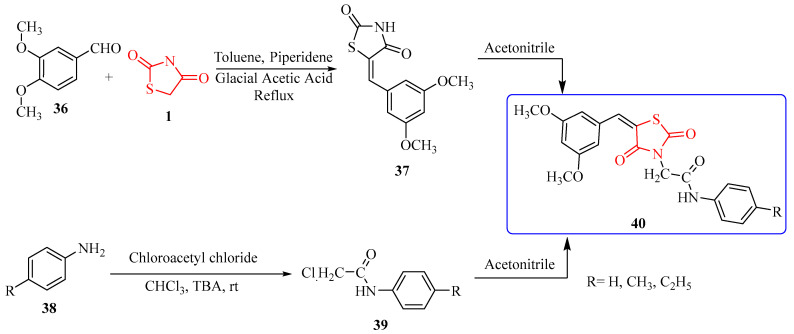
Synthesis of 3,5-diubstituted thiazolidinedione ring system.

**Figure 9:**
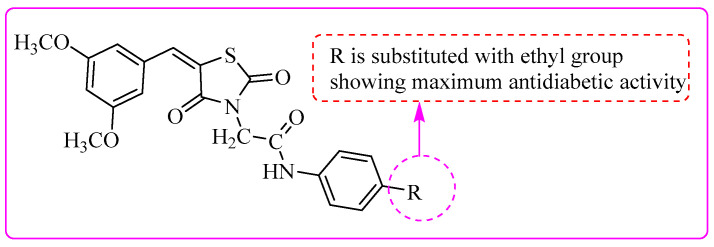
SAR of 3,5-diubstituted thiazolidinedione ring system.

In search of new antidiabetic agents, heterocyclic compounds containing 3,5-substituted thiazolidinedione moieties **40** were synthesized through a concise three-step reaction process, as described in Scheme 7. The synthesis involved Knoevenagel condensation at the 5th position of the 3,5-substituted thiazolidinedione ring system. The synthesized derivatives were subjected to evaluation for their in vivo antidiabetic activity against diabetes-induced Wistar rats and in vitro activity against α-amylase, α-glucosidase, and glucose uptake by yeast cells. The compounds substituted with the ethyl group were predicted to have the greatest effect out of the compounds **40**, showing interactions with targets which exhibited potential binding patterns against the active sites of target α-amylase and α-glucosidase with modulating AMY2A, GAA, PPARG, PIK3CA, PRKCB, INSR, and PRKCB signaling pathways, as described in Figure 9 [56]. The ethyl-substituted compounds showed in vitro α-amylase and α-glucosidase inhibitory activity, with IC50 values of 86.06 ± 1.1 μM and 74.97 ± 1.23 μM, compared to the standard drug acarbose (IC50 values of 26.89 ± 3.12 μM and 29.25 ± 0.15 μM). They also demonstrated 58.23 ± 0.13% glucose uptake in vitro and significantly reduced blood glucose levels in vivo (114 ± 1.17 mg/dL, *p* < 0.001), which was comparable to the effect of pioglitazone (102.2 ± 0.79 mg/dL).

**Scheme 8:**
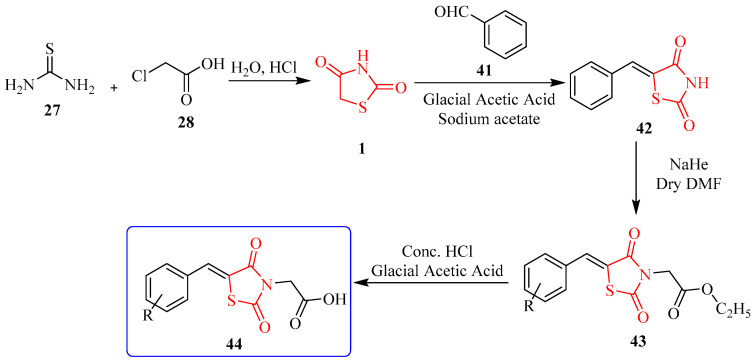
Synthetic scheme of [5-(3,4-substituted)benzylidine2,4-thiazolidinedione,5-(3,4,5 trimethoxy)benzylidine2,4-thiazolidenedione] and derivatives.

**Figure 10:**
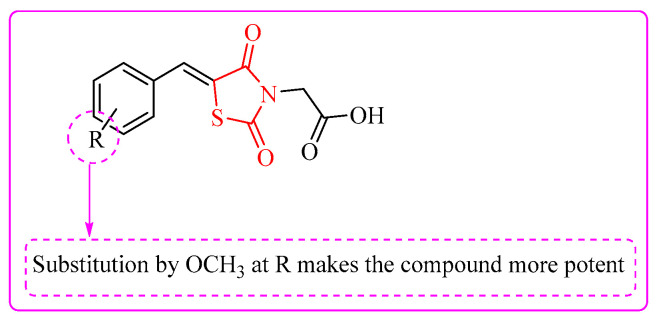
SAR of [5-(3,4-dimethoxy)benzylidine2,4-thiazolidinedione,5-(3,4,5 trimethoxy)benzylidine2,4-thiazolidenedione].

**Scheme 9:**
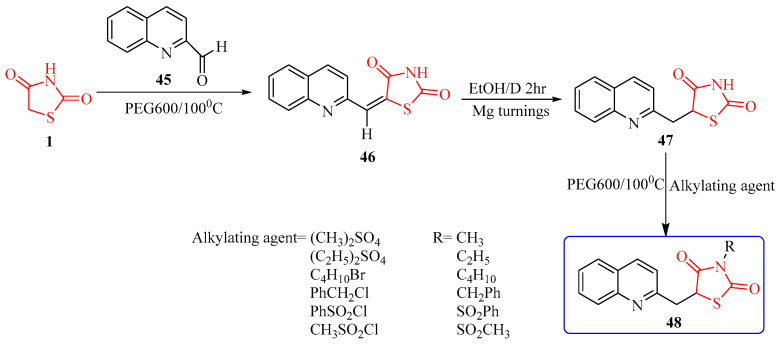
Synthesis of (N-substituted-5-quinolidenyl)-2, 4-thiazolidiendione.

Datar et al. synthesized a new series of thiazolidinediones **44** via reaction between thiazolidinedione **1** along with several benzaldehyde derivatives **41** using Scheme 8. The compound **44** was screened for antidiabetic activity using the SLM model, and the compound found with methoxy substitution was found to be more active [5-(3,4-dimethoxy)benzylidine2,4-thiazolidinedione,5-(3,4,5 trimethoxy)benzylidine2,4-thiazolidenedione], as mentioned in Figure 10 [51].

(N-Substituted-5-quinolidenyl)-2,4-thiazolidinedione **48** was synthesized using polyethylene glycol-600 as an alkylating agent. The resulting mixture was then heated to complete the reaction. This step further led to the synthesis of the end product 5-[(quinolin-2-yl)methyl]-1,3-thiazolidine-2,4-dione **46** by PEG-600 at 100 °C (Scheme 9). All the compounds were tested for antidiabetic activity. Compounds synthesized and assessed with conventional drugs are shown to be highly potent [57].

**Scheme 10:**
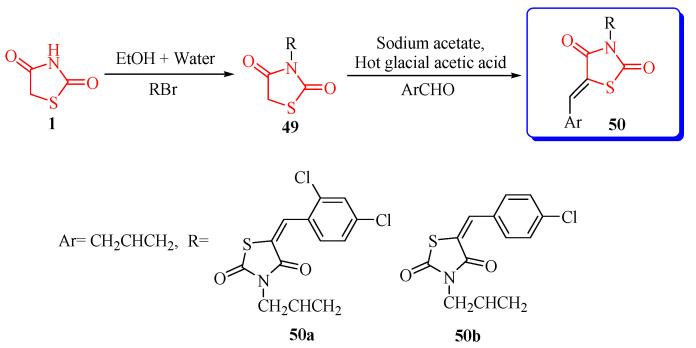
Synthetic strategy of 5-arylidene derivatives of thiazolidinedione.

**Figure 11:**
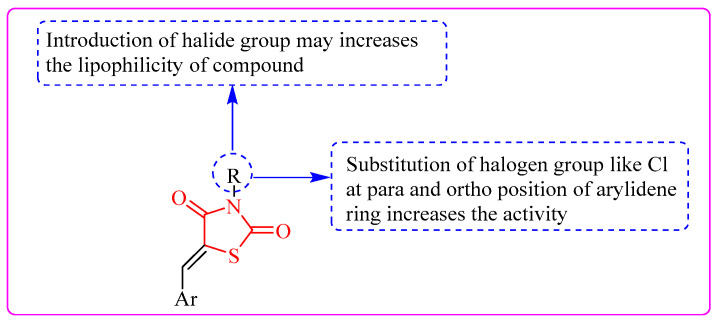
SAR of 5-arylidene derivatives.

Synthesis of 5-arylidene derivatives **50** was performed by the inclusion of different aromatic aldehydes in glacial acetic acid using thiazolidinedione **1** as the starting material in ethanol and water, as represented in Scheme 10. All the compounds in this scheme were tested for their antidiabetic potential. -Allyl-5-(2, 4-dichlorobenzylidine)thiazolidine-2, 4-dione synthesized compounds show effective activity when using the reference drug rosiglitazone. SAR analysis of structural modifications can influence glucose-lowering effects, insulin sensitivity, or enzyme inhibition (like α-glucosidase or DPP-4), and details are illustrated in Figure 11 [58].

**Scheme 11:**
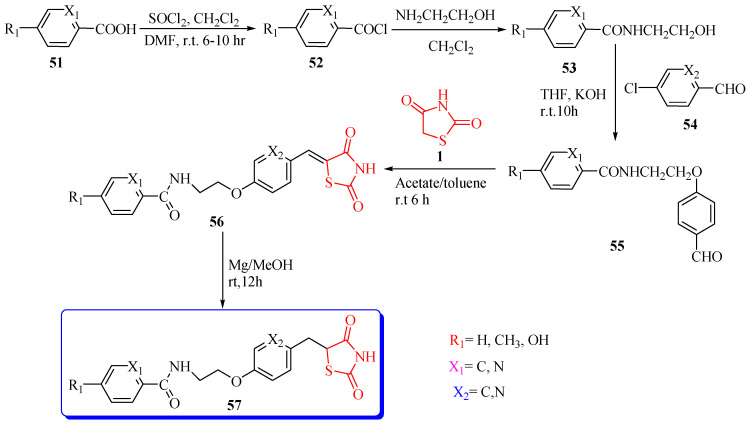
Synthesis of N-(2–(4-((2, 4-dioxothiazolidin-5-yl) methyl) substituted) ethyl) benzamide.

**Figure 12:**
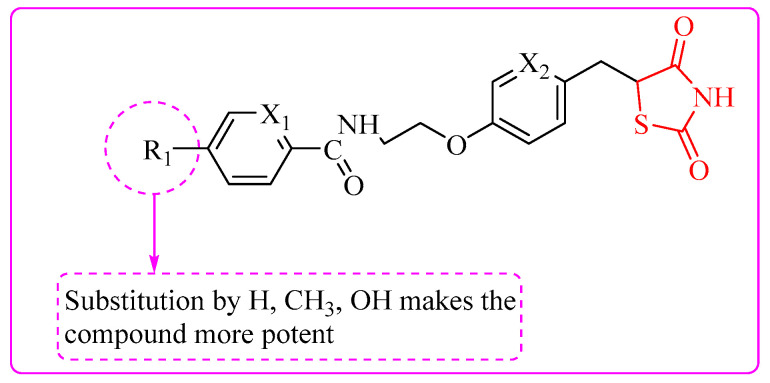
SAR of N-(2–(4-((2, 4-dioxothiazolidin-5-yl) methyl) substituted) ethyl) benzamide derivatives.

In the given study, the synthesis of N-(2–(4-((2, 4-dioxothiazolidin-5-yl) methyl) substituted) ethyl) benzamide **56** derivatives was performed, with the help of magnesium and methanol at room temperature, using substituted benzoic acids **52** with SOCl_2_ in dimethylformamide under specific temperature conditions for 6–10 h. All these compounds were evaluated for antidiabetic activity, and the synthesized compound N-(2-(4-((2, 4-dioxothiazolidin-5-yl) methyl) phenoxy) ethyl)-4-methylbenzamide shows effective activity compared with rosiglitazone. The synthetic procedure is discussed in Scheme 11, with SAR analysis shown in Figure 12. On examination, due to the functional groups like R1, X1, or X2, a medicinal chemist can enhance the selectivity and physicochemical characteristics. The compounds substituted with H, CH_3_, or OH groups can increase activity [59].

**Scheme 12:**
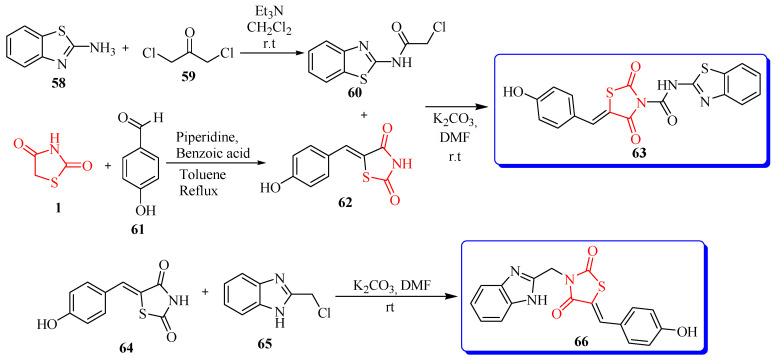
Synthesis of thiazolidinediones derivatives.

**Figure 13:**
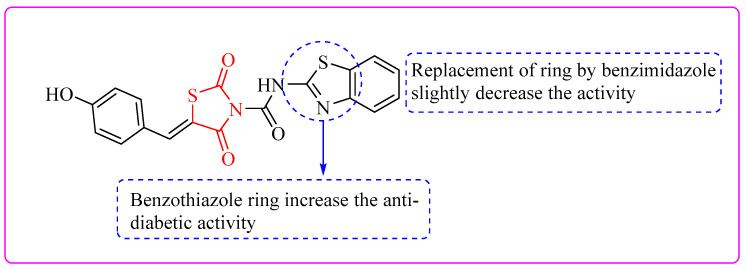
SAR of thiazolidinediones containing benzthiazoles.

In this review, the synthesis of N-(1, 3-benzo[d]thiazol-2-yl)-2-[(5Z)-5-(4-hydroxybenzylidene)-2,4-dioxo-1,3-thiazolidin-3-yl] acetamide **63** and (5Z)-3-(1H-benzo[d]imidazol-2-ylmethyl)-5-(4-hydroxybenzylidene)-1,3-thiazolidine-2,4-dione **66** have been completed by potassium carbonate in dimethylformamide at room temperature. This two-step reaction was completed with the help of an appropriated route of synthesis, in which the first step started from benzothiazol-2-ylamine **58** and another step started from thiazolidinedione **1,** as described in Scheme 12 with the SAR analysis given in Figure 13. All synthesized compounds were taken into antidiabetic studies and most of the compounds were found to have highly potent compounds. The SAR shown in Figure 13 indicates that the benzothiazole ring enhances antidiabetic activity, while replacing it with a benzimidazole ring slightly reduces the activity [60].

**Scheme 13:**
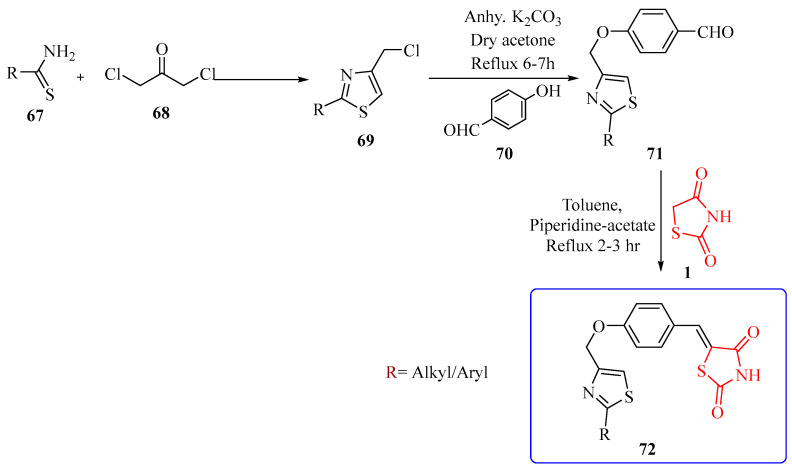
Schematic representation for synthesis of 5-[4-(2-methyl/phenylthiazol-4-ylmethoxy) benzylidene]-thiazolidine-2, 4-diones.

**Figure 14:**
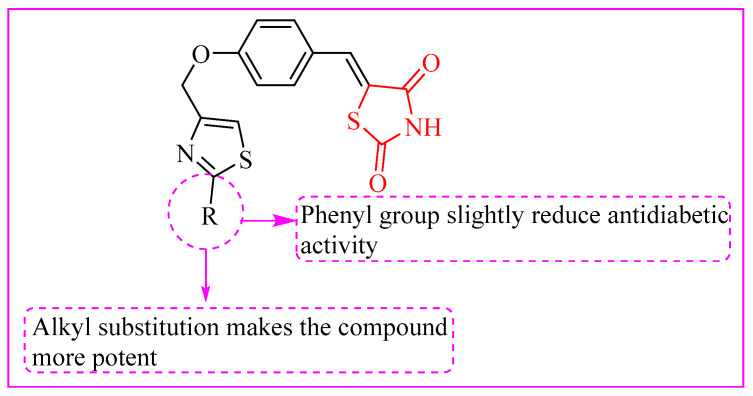
SAR of 5-[4-(2-methyl/phenylthiazol-4-ylmethoxy) benzylidene]-thiazolidine-2, 4-diones.

Synthesis of 5-[4-(2-methyl/phenylthiazol-4-ylmethoxy) benzylidene]-thiazolidine-2, 4-diones **72** has been completed from the starting material thioformamides **67** and 1, 3-Dichloro-propan-2-one **68,** proceeding to the formation of the final compounds after passing through the different appropriate routes of the chemical reaction (Scheme 13). 5-[4-(2-{[5-(4-Methoxyphenyl)-4H-1,2,4-triazol-3-yl]thio}ethoxy) benzylidene]-1,3-thiazolidine-2,4-dione of synthesized and assessed compounds shows effective potency when compared to pioglitazone, and the SAR is discussed in Figure 14 [61].

**Scheme 14:**
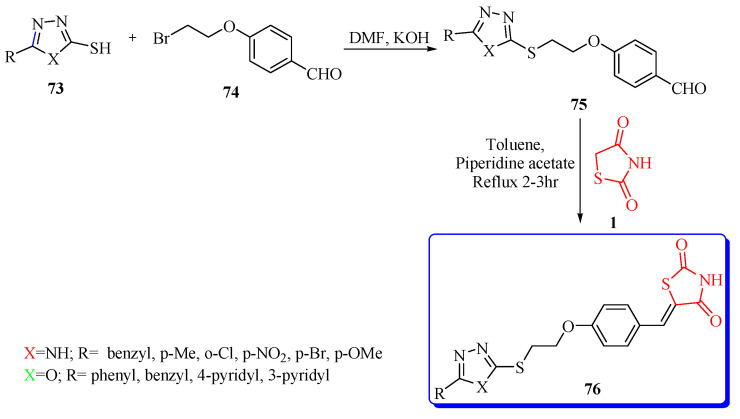
Synthesis of 4-{2-[(5-aryl-4H-1, 2, 4-triazole-3-yl) thio] ethoxy} benzylidene-thiazolidinediones derivatives.

**Figure 15:**
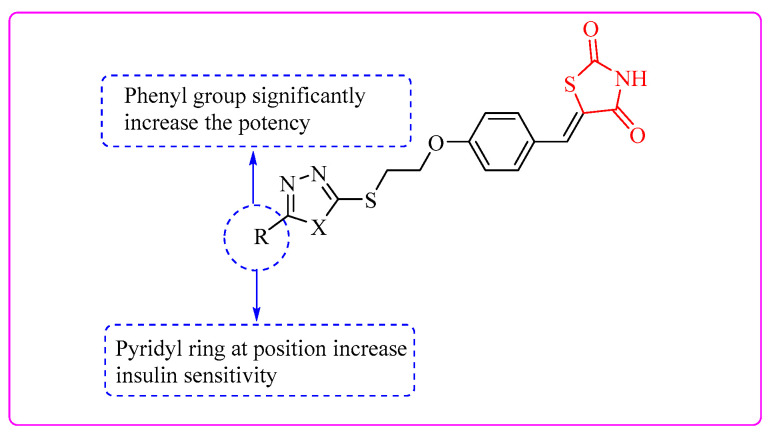
SAR of 4-{2-[(5-aryl-4H-1, 2, 4-triazole-3-yl) thio] ethoxy} benzylidene-thiazolidinediones.

Synthesis of 4-{2-[(5-aryl-4H-1, 2, 4-triazole-3-yl) thio] ethoxy} benzylidene-thiazolidinediones **76** has been completed with the help of toluene and piperidine acetate under reflux for 2–3 h via the synthetic route of 4-[2-(4H-[1,2,4]Triazol-3-ylsulfanyl)-ethoxy]-benzaldehyde **75** initiated from 4H-[1,2,4]Triazole-3-thiol **73** and 4-(2-Bromo-ethoxy)-benzaldehyde **74**. Evaluation for the antidiabetic activity has been performed and all the compounds were tested for the on-basis activity with reference drug pioglitazone. Consequently, 5-(4-{2-[(5-Pyridin-3-yl-1,3,4-oxadiazol-2-yl)thio]ethoxy}benzylidene)-1,3-thiazolidine-2,4-dione and 5-(4-{2-[(5-Pyridin-4-yl-1,3,4-oxadiazol-2-yl)thio]ethoxy}benzylidene)-1,3-thiazoli dine-2,4-dione synthesized compounds shows high potency [61]. The synthesis is described in Scheme 14, and the SAR analysis is in Figure 15. The phenyl ring increases the potency, while the pyridyl ring increases insulin sensitivity.

**Scheme 15:**
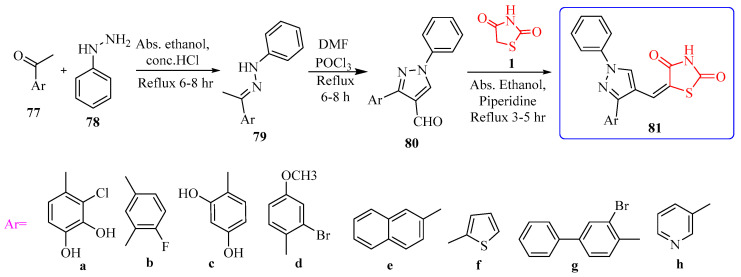
Synthesis of 2, 4-thiazoldinedione derivatives.

**Figure 16:**
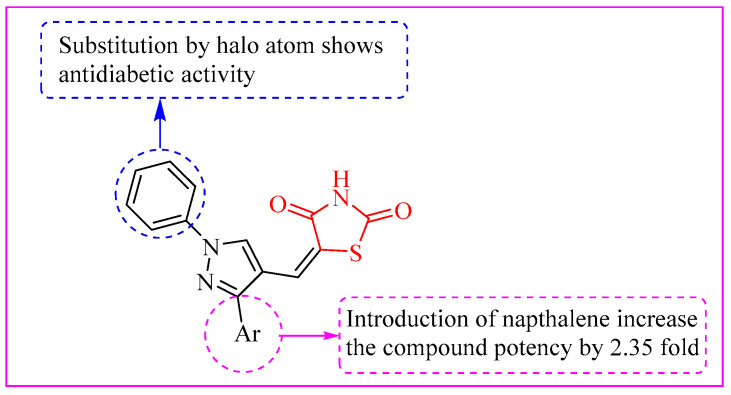
SAR of 2, 4-thiazoldinedione derivatives.

In this reaction 2, 4-thiazoldinedione derivatives **81(a–h)** were synthesized, initiated from aldehydes **77** and phenyl hydrazine **78** in absolute alcohol and conc. HCl under reflux for 6–8 h. Further, this step gives the pioneer compound for the end product synthesis, using 1-Phenyl-1H-pyrazole-4-carbaldehyde as the intermediate **80**. The intermediate compound reacts with piperidine in ethanol, leading to incorporation of the thiazolidinedione nucleus, as illustrated in Scheme 15. Most of the synthesized compounds were evaluated for activity. Among them, 5-((3-(Naphthalen-2-yl)-1-phenyl-1H-pyrazol-4-yl)methylene)-thiazolidine-2,4-dione exhibited high potency, comparable to the standard drugs rosiglitazone and pioglitazone. The SAR is shown in Figure 16 [62].

**Scheme 16:**
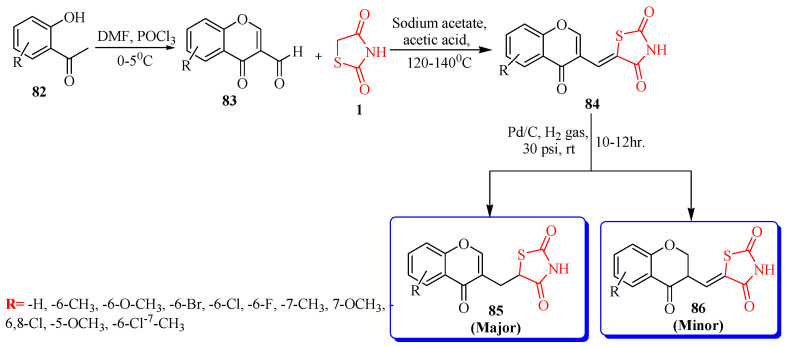
Schematic illustration of [5-(substituted chromen-3-yl) methyl] thiazolidine2, 4-diones.

**Figure 17:**
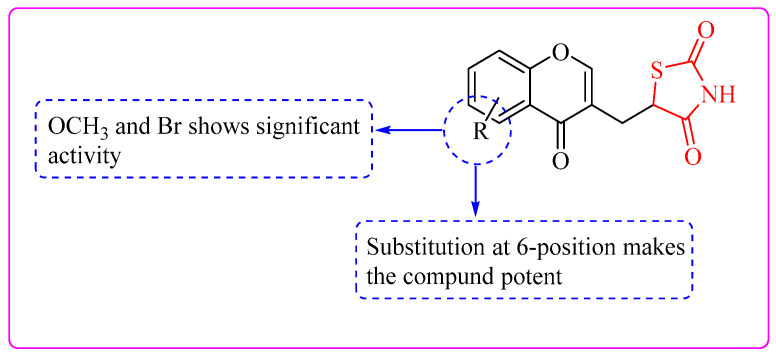
SAR of [5-(substituted chromen-3-yl) methyl] thiazolidine2, 4-diones.

In this study of the research, synthesis of [5-(substituted chromen-3-yl) methyl] thiazolidine-2,4-diones **85** and **86** was reported with the help of the Vilsmeier–Hack reaction initiated from 1-(2-hydroxy-phenyl)-ethanone **82** in the presence of DMF and POCl_3_ under the coolest temperature conditions. Further, the above reaction reacted with sodium acetate in acetic acid to give the end product as major and minor (Scheme 16). The synthesized compounds had maximal and moderate antidiabetic activity, but the most active compounds were 5[(6-methoxy-4-oxo-4H-chromen-3yl) methyl] thiazolidine-2, 4-dione, and 5[(6-chloro-4-oxo-4H-chromen-3yl) methyl] thiazolidine-2: 4-dione compared with standard drug pioglitazone and the detailed SAR is described in Figure 17. The substitution with different groups at the R position like OCH_3_ and Br shows significant activity, while substitution at the 6th position increases potency [63].

**Scheme 17:**
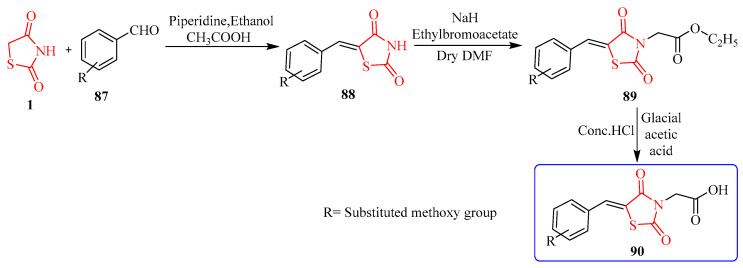
Synthesis of [5-(substituted benzylidene)-2, 4-dioxo-thiazolidin-3-yl]-acetic acids.

**Figure 18:**
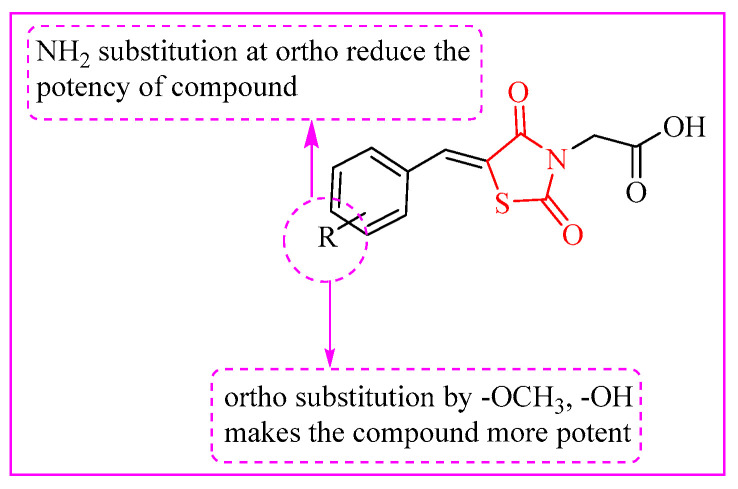
SAR of [5-(substituted benzylidene)-2, 4-dioxo-thiazolidin-3-yl]-acetic acids.

2,4-thiazolidinedione **1** and benzaldehyde derivative **87** were reacted in piperidine with ethanol as the reflux solvent, using thiazolidinedione as the starting material, resulting in the formation of [5-(substituted benzylidene)-2,4-dioxo-thiazolidin-3-yl] -acetic acid ethyl ester **89** using anhydrous DMF. Additionally, all of the above reactants yield the final product [5-(substituted benzylidene)-2, 4-dioxo-thiazolidin-3-yl]-acetic acids **90** (Scheme 17), with the structure–activity relationship depicted in Figure 18. The majority of the compounds were successfully synthesized; among them, 5-(3,4-dimethoxy)benzylidine-2,4-thiazolidinedione and 5-(3,4,5-trimethoxy)benzylidine-2,4-thiazolidinedione exhibit much more activity compared to pioglitazone [64].

**Scheme 18:**
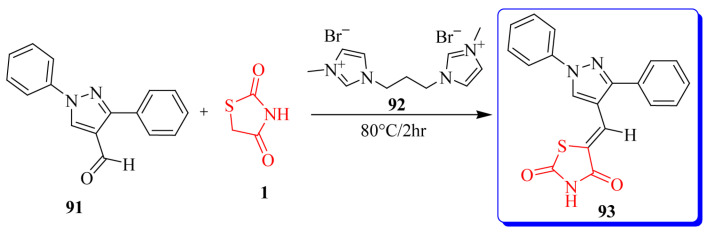
A synthetic route for synthesis of 1,3-diphenylpyrazolinyl-2,4-thiazolidinediones.

**Figure 19:**
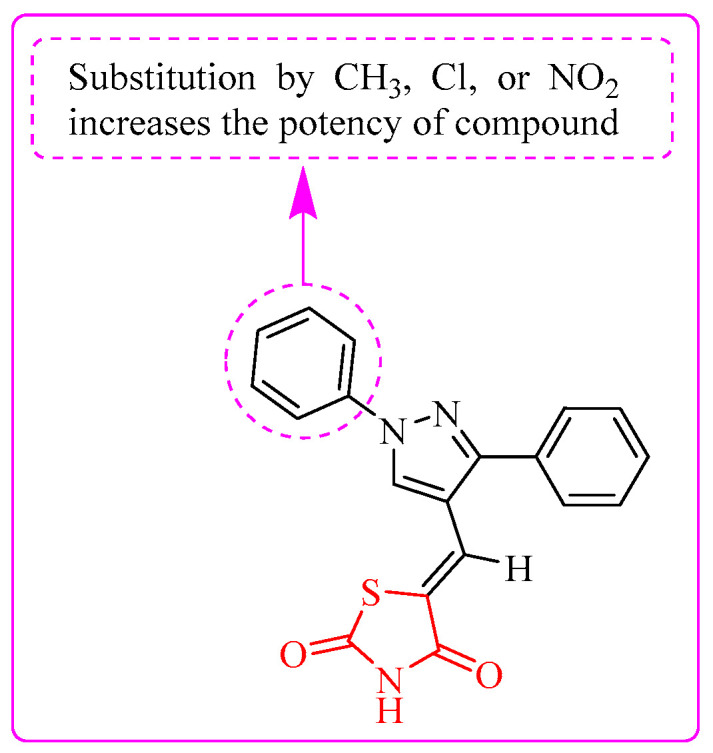
SAR of 1,3-diphenylpyrazolinyl-2,4-thiazolidinediones.

Jawale et al. reported the synthesis of 1,3-diphenylpyrazolinyl-2,4-thiazolidinediones **93** via the condensation of **91** with **1**, using 3-methyl-1-[3-(methyl-1H-imidazolium-1-yl)propyl]-1H-imidazolium dibromide **92** as a di-cationic ionic liquid at 80 °C for 2 h. The detailed procedure is described in Scheme 18, along with the SAR in Figure 19. Results indicate that the synthesized product **93** is biologically active and acts as a precursor of antidiabetic drugs. Substitution with CH_3_, Cl, or NO_2_ on the pyrazoline nucleus greatly enhances activity. Presence of both the core ring and the nature of substituents play a crucial role in optimizing biological effects [65].

**Scheme 19:**
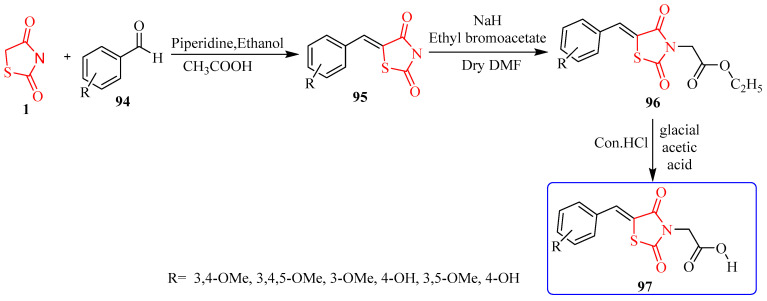
A schematic synthesis for substituted benzylidene thiazolidinedione acetic acid derivatives.

**Figure 20:**
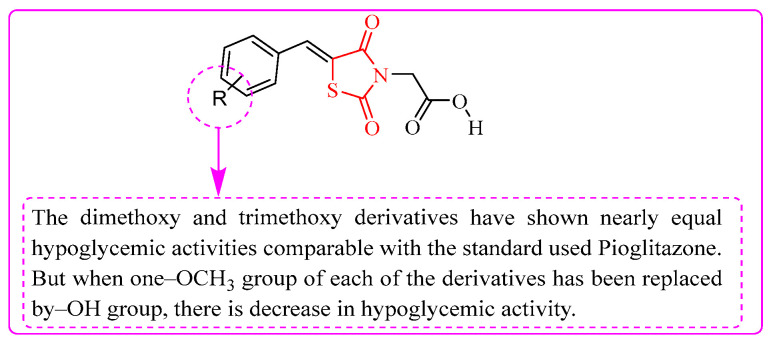
SAR of substituted benzylidene thiazolidinedione acetic acid derivatives.

Prashantha et al. reported the synthesis of substituted benzylidene thiazolidinedione acetic acid derivatives **97** through the reaction of thiazolidine-2,4-dione **1** and a benzaldehyde derivative **94**, which undergo Knoevenagel condensation to yield benzylidene thiazolidinedione **95**. Subsequent N-alkylation with ethyl bromoacetate produces alkyl 2,4-dioxothiazolidin-3-ethyl ester **96**, which is then converted to the acid derivative **97** using concentrated HCl. This is predicted to exhibit significant antidiabetic properties. Their impact on hypoglycemic activity was assessed using a sucrose-loaded model. The findings demonstrate that the dimethoxy and trimethoxy compounds possess hypoglycemic properties comparable to the standard reference, pioglitazone. However, substituting one OCH_3_ group with an –OH group results in a decrease in hypoglycemic activity, as reported in Scheme 19 and Figure 20 for SAR analysis for compound **97**. The replacement of OCH_3_ group with an OH group in the shown compound reduces its activity, which helps in designing more effective and safer drugs by guiding structural optimization [66].

### 2.2. Anticancer Agents

Multiple TZDs have shown potential anticancer effects, and their structural frameworks have been extensively studied for therapeutic use. This presentation covers novel molecules developed for potential use as anticancer agents.

**Scheme 20:**
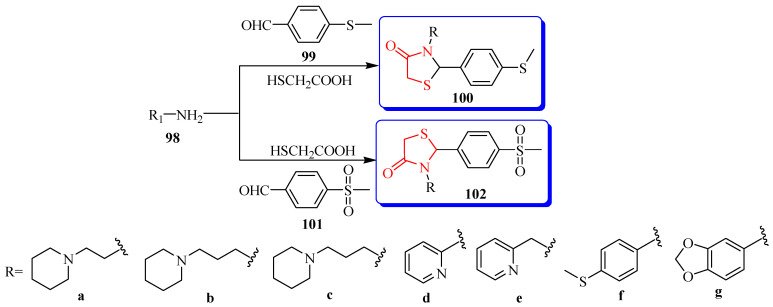
Synthetic strategy for thiazolidin-4-ones and their derivatives.

**Figure 21:**
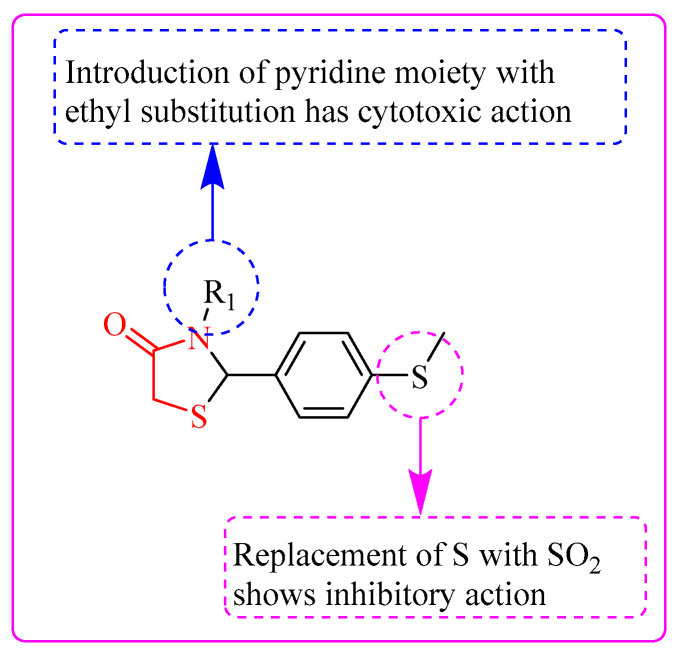
SAR of thiazolidin-4-ones and their derivatives.

Da Silva and coworkers evaluated the anti-glioma activity and cytotoxicity of thiazolidin-4-ones. In this procedure, primary amine **98**, aldehydes **99** or **101,** and mercaptoacetic acid were reacted via one-pot MCR in the presence of BF3 and p-toluenesulfonic acid (PTSA) and formed derivatives of thiazolidin-4-one. Da Silva and colleagues assessed the anti-glioma activity and cytotoxic effects of thiazolidin-4-ones. This procedure involves the reaction of primary amine **98**, aldehydes **99** or **101,** and mercaptoacetic acid in a one-pot multicomponent reaction (MCR) utilizing boron trifluoride (BF_3_) and p-toluenesulfonic acid (PTSA), resulting in the formation of various thiazolidin-4-one derivatives **100** and **102**, as shown in Scheme 20**: t**he SAR activity is depicted in Figure 21. Among all synthesized derivatives, **100**b, **100**e, **100**g, and **100**e exhibited potent antitumor effects against reference cells. Introduction of an ethyl-pyridine group increases cytotoxicity, while replacing sulfur with SO_2_ enhances inhibition [67].

**Scheme 21:**
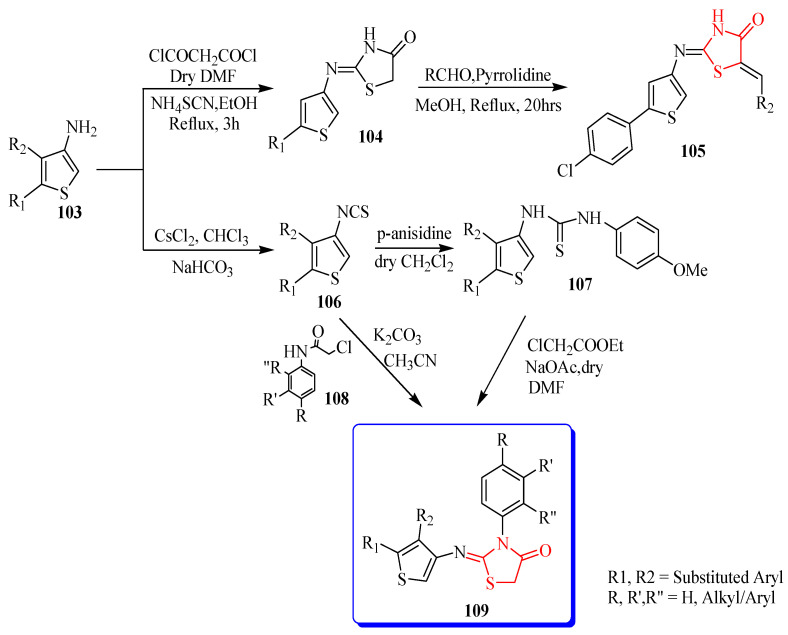
Synthesis of 2-heteroaryliino-1,3-thiazolidine-4-one derivatives.

**Figure 22:**
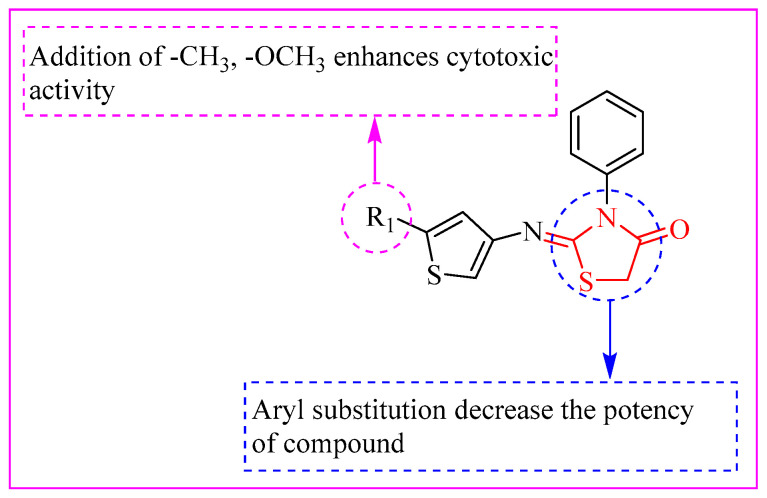
SAR of 2-heteroaryliino-1,3-thiazolidine-4-ones.

The synthesis of 2-heteroaryliino-1,3-thiazolidine-4-ones **105** and **109** using 3-amino thiophenes, chloroacetyl chloride, and ammonium thiocyanate, along with different solvents and catalysts, was reported by Revelant et al. in**Scheme 21**. Introducing an aryl group at the N-3 position of the thiazolidinone ring significantly diminished the anticancer efficacy, while adding a benzylidene group at position 5 is crucial. It was observed that compounds with p-methoxybenzylidene**,** p-methylbenzylidene, p-dimethylaminobenzylidene, and 3,4-dimethoxybenzylidene exhibited the effect on HT29 cell growth inhibition. The SAR of 2-methoxyphenylino-1,3-thiazolidine-4-one shows increased cytotoxic activity, as depicted in**Figure 22 [68]**.

**Scheme 22:**
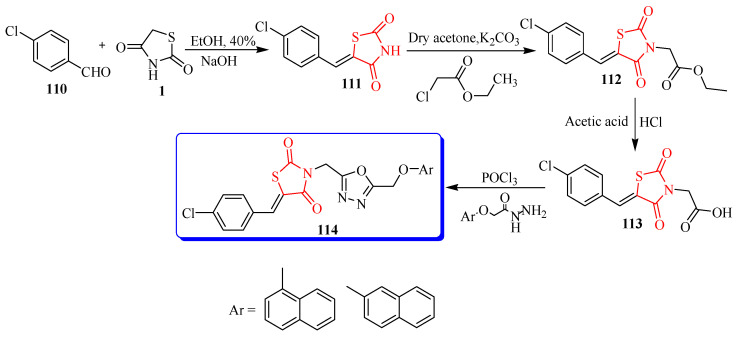
Synthetic scheme of thiazolidinedione derivatives.

**Figure 23:**
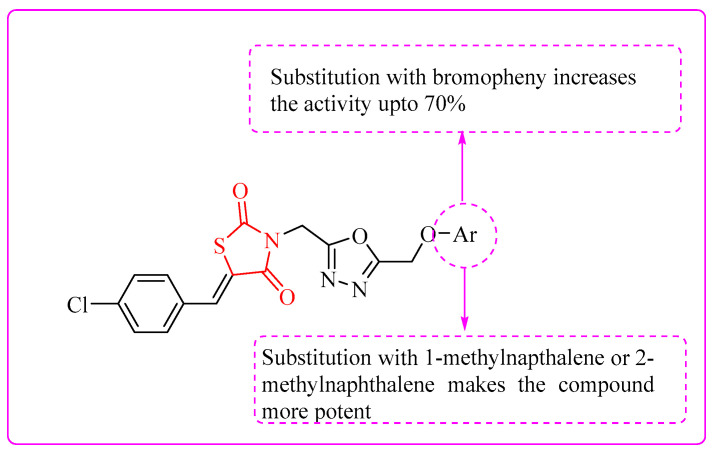
SAR of thiazolidinedione derivatives.

Compound **111** was produced by the Knoevenagel condensation of thiazolidinedione **1** with 4-chlorobenzaldehyde **110** in the presence of 40% aqueous NaOH and absolute ethanol. Then, using potassium carbonate as a base, this molecule was alkylated with ethyl chloroacetate in acetone. The intermediate **113** was obtained by subsequent acidic hydrolysis, as mentioned in Scheme 22. By refluxing intermediate **113** with hydrazides of different phenoxyacetic acids in phosphoryl chloride (POCl_3_), the compound **114** and its derivatives were synthesized. In this study the results of PPAR-γ transactivation indicated that compounds substituted with 1-nphthyl and 2-naphthyl show maximum potency, enhancing PPAR-γ gene expression by 2.2- and 2.4-fold, respectively. These compounds also demonstrated notable cytotoxicity, with IC_50_ values ranging from 1.4 to 4.5 μM against MCF-7 cells and 1.8 to 8.4 μM against HCT-116 cells. The SAR in Figure 23 states that bromobenzene, methylnaphthalene, naphthyl, or coumarinyl groups significantly enhance the compound’s activity [69].

**Scheme 23:**
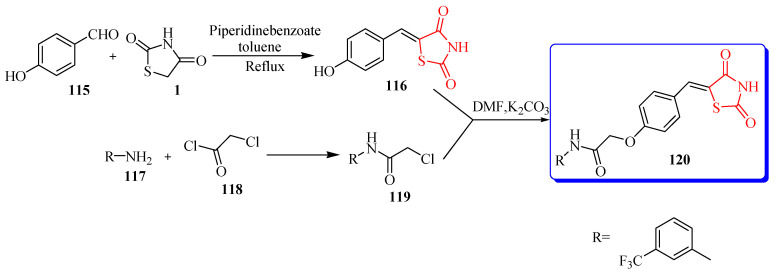
Synthetic scheme of 5-benzylidene-2, 4-thiazolidinediones synthesis.

**Figure 24:**
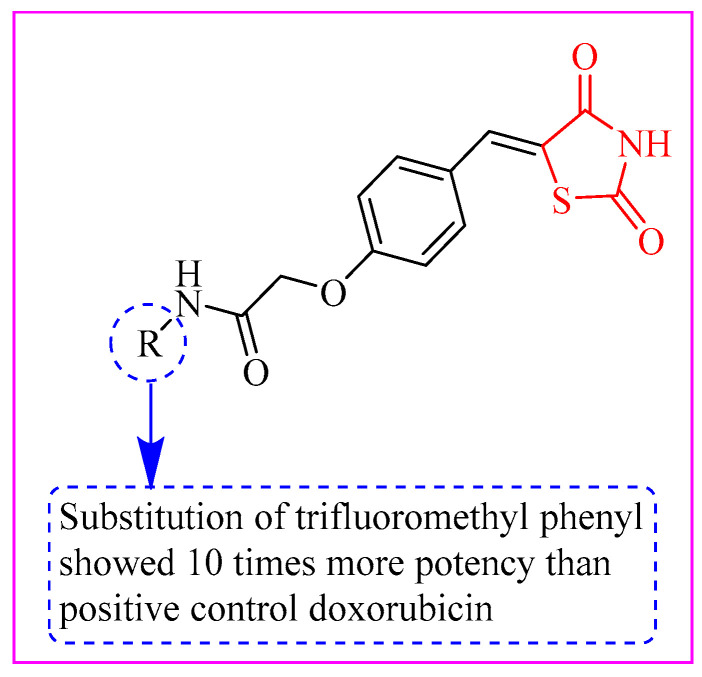
SAR of 5-benzylidene-2, 4-thiazolidinediones.

A series of 5-benzylidene-2, 4-thiazolidinediones **120** were synthesized through the condensation of 5-(4-hydroxybenzylidene)-2, 4-thiazolidinedione **116** with chloroacetylated heteroaromatic amines or heterocyclic nitrogen-containing compounds **116**. Initially, 5-(4-hydroxybenzylidene)-2, 4-thiazolidinedione **116** was synthesized through a Knoevenagel condensation reaction involving 4-hydroxybenzaldehyde **115** and 2, 4-thiazolidinedione **1**. Secondly, chloroacetylated moieties **119** were synthesized through the acetylation of amines **117** with chloroacetyl chloride **118** under basic conditions, as illustrated in Scheme 23, with the structure–activity relationship (SAR) presented in Figure 24. The synthesized compounds were assessed for their anticancer activity across a targeted in vitro panel of seven cell lines: HEPG2 (hepatoma), HOP62 (lung cancer), KB (nasopharyngeal cancer), MCF7 (breast cancer), K562 (leukemia), and PC3 (prostate cancer). All derivatives demonstrate high potency against selective cancer cell lines [70].

**Scheme 24:**
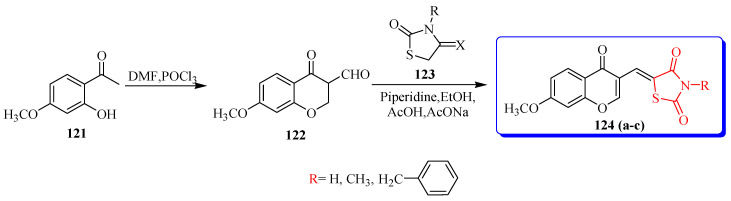
Synthetic framework for chromonylthiazolidines **169(a–c)**.

**Figure 25:**
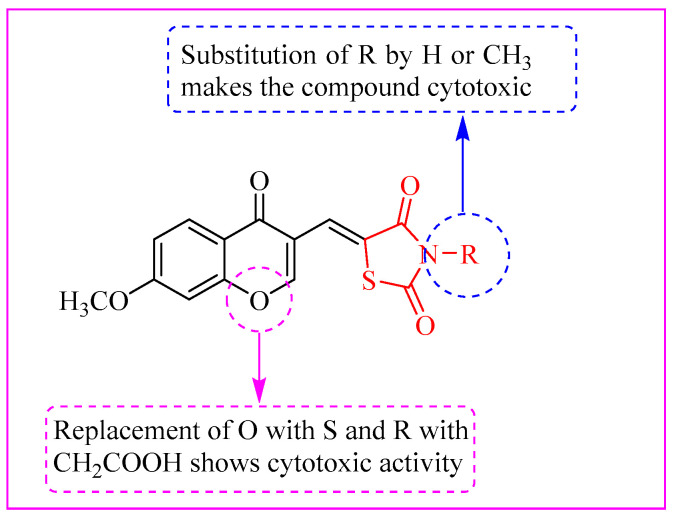
SAR of chromonylthiazolidines **169(a–c)**.

According to the literature, the synthesis of chromonylthiazolidines **124(a**–**c)** was performed in two steps. First, preparation of 3-formyl-7-methoxychromone **122** through the Vilsmeier–Haack reaction of 2-hydroxy-4-methoxybenzaldehyde **121** with dimethylformamide and phosphorus oxychloride. Then, in the presence of an appropriate base catalyst, such as piperidine, anhydrous sodium acetate, or glacial acetic acid, 3-formyl-7-methoxychromone **122** and thiazolidine derivatives **123** were reacted to obtain chromonylthiazolidines **124(a**–**c)** (Scheme 24). Among all the derivatives, 5-((7-Methoxy-4-oxo-4H-chromen-3-yl) methylene) thiazolidine-2,4-dione and 5-((7-Methoxy-4-oxo-4H-chromen-3-yl)methylene)-3-methyl-thiazolidine-2,4-dione show the most selective cytotoxic effects towards the tested cancer cell lines, and the SAR is described in Figure 25. The substitution of the R group with H or CH_3_ in the shown compound increases cytotoxicity [71].

**Scheme 25:**
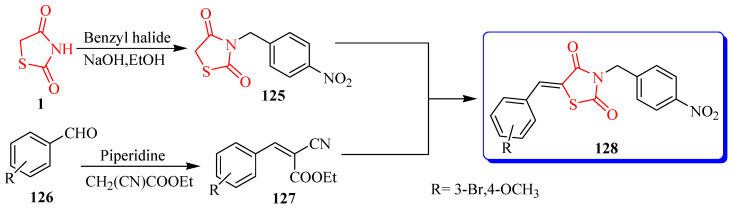
Synthesis of 3-(4-nitrobenzyl)-thiazolidine-2,4-dione and derivatives.

**Figure 26:**
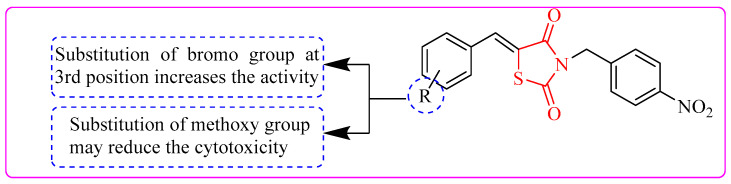
SAR of 3-(4-nitrobenzyl)-thiazolidine-2,4-dione and derivatives.

Rego et al. documented the synthesis of 3-(4-nitrobenzyl)-thiazolidine-2,4-dione **128** through the condensation of thiazolidine-2,4-dione **1** with 4-nitrobenzyl bromide, utilizing KOH in ethanol as a catalyst. An equimolar mixture of 3-(4-nitrobenzyl)-thiazolidine-2,4-dione **125** and a suitably substituted ethyl-2-cyano-3-phenylacrylate **127** in ethanol with piperidine was stirred under reflux for 4 h and subsequently cooled to room temperature, as depicted in Scheme 25, with the structure–activity relationship presented in Figure 26. Among the three newly synthesized thiazolidinediones, (5Z)-5-(3-bromo-benzylidene)-3-(4-nitrobenzyl)-thiazolidine-2,4-dione had the most significant activity as it exhibited preferential cytotoxicity towards leukemia, lymphoma, hepatocarcinoma, and glioblastoma cell lines while sparing normal cells from toxicity [72].

### 2.3. Antimicrobial Activity

TZDs demonstrate considerable antimicrobial activity, and their synthesis processes are extensively described. This presentation presents new molecules intended for development as antimicrobial agents.

**Scheme 26:**
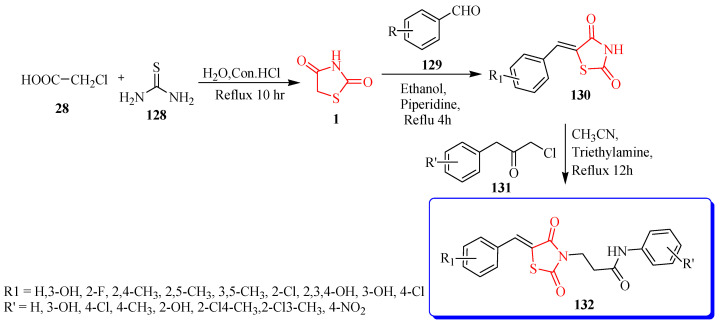
Synthesis of N-3-dialylaminomethyl-5-benzylidene-2, 4-thiazolidinediones.

**Figure 27:**
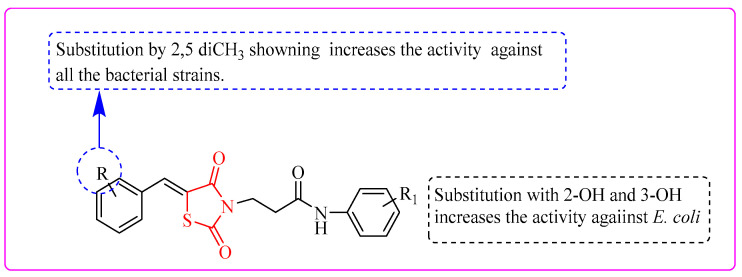
SAR of N-3-dialylaminomethyl-5-benzylidene-2, 4-thiazolidinediones.

A series of novel substituted 2-(5-benzylidene-2,4-dioxothiazolidin-3-yl)-N-(phenyl)propanamides **132** were created by using 5-benzylidene-2,4-thiazolidinediones **130** (0.01 mol) and the corresponding substituted N-phenylpropanamides **131,** as outlined in Scheme 26. All synthesized compounds were assessed for in vitro antibacterial activity against Gram-positive bacteria, Staphylococcus aureus (NCIM 2122) and Bacillus subtilis (MTCC 121), and Gram-negative bacteria, *Escherichia coli* (MTCC 118), *Pseudomonas aeruginosa* (MTCC 647), *Salmonella typhi* (NCIM 2501), Klebsiella pneumonia (MTCC 3384), and fungi: Candida albicans (MTCC 227), and Aspergillus niger (NCIM 1056), utilizing the two-fold serial dilution technique: ciprofloxacin and fluconazole were used as the standard for antibacterial and antifungal efficacy, respectively. The SAR is discussed in Figure 27 and it indicates that substitution with 4-nitro, chloro, or fluoro groups enhances activity, while bromo, methylnaphthalene, naphthyl, and coumarinyl groups significantly increase overall activity [73].

**Scheme 27:**
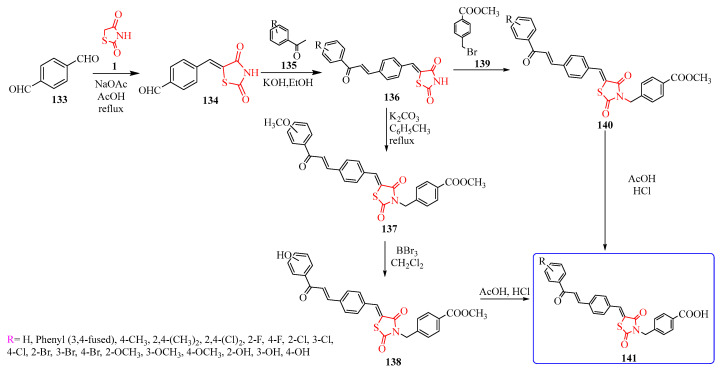
Synthetic scheme of 4-((5-((Z)-4-((E)-3-(substitutedphenyl)-3-oxoprop-1-en-1-yl)benzylidene)-2,4-dioxothiazolidin-3-yl)methyl)benzoic acid.

**Figure 28:**
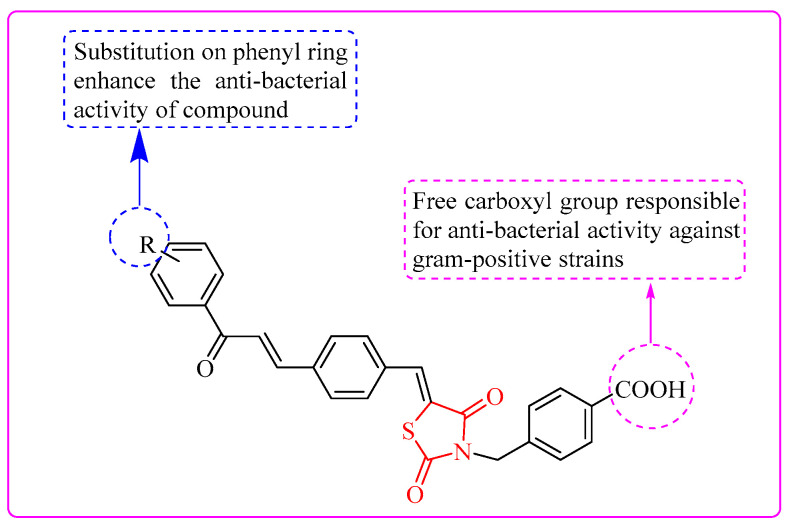
SAR analysis for 4-((5-((Z)-4-((E)-3-(substitutedphenyl)-3-oxoprop-1-en-1-yl)benzylidene)-2,4-dioxothiazolidin-3-yl)methyl)benzoic acid.

In this study, Knoevenagel condensation takes place between terephthalic aldehyde **179** and thiazolidinedione **1** to form (Z)-4-((2,4-Dioxothiazolidin-5-ylidene) methyl) benzaldehyde **180**. Further, synthesis of (Z)-5-(4-((E)-3-oxo-3-phenylprop-1-en-1-yl) benzylidene) thiazolidine-2,4-dione **182** was performed via Claisen–Schmidt condensation between **180** and acetophenones **181**. By refluxing **182** for 2 h in toluene with methyl 4-(bromomethyl) benzoate **185,** using potassium carbonate as a base, the final derivative 4-(Substituted)-benzylidene) thiazolidine-3-yl) methyl) benzoic acid **187** (Scheme 27) was prepared and evaluated for its antimicrobial activity against Gram-positive (*Staphylococcus aureus*) and -negative strains (*Escherichia coli*). Most of the synthesized compounds are shown to be highly potent. It demonstrates that the substituted derivatives, specifically those with hydrogen, 2-chloro, 2-bromo, 2-methoxy, and 2-hydroxy groups, exhibit increased potency, showing MIC values ranging from 0.5 to 4 mg/mL against six different *Gram-positive* bacteria. In SAR the presence of an electron donating group (EDG) like CH_3_ increases electron density on the aromatic ring, while an electron withdrawing group like Cl and Br stabilizes the molecule (Figure 28) [74].

**Scheme 28:**
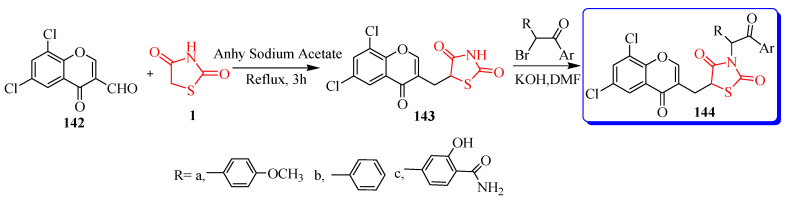
Schematic presentation of 5-((6,8-dichloro-4-oxo-4H-chromen-3-yl)methyl)-3-(1-substituted-2-oxo-2-arylethyl)thiazolidine-2,4-dione synthesis.

**Figure 29:**
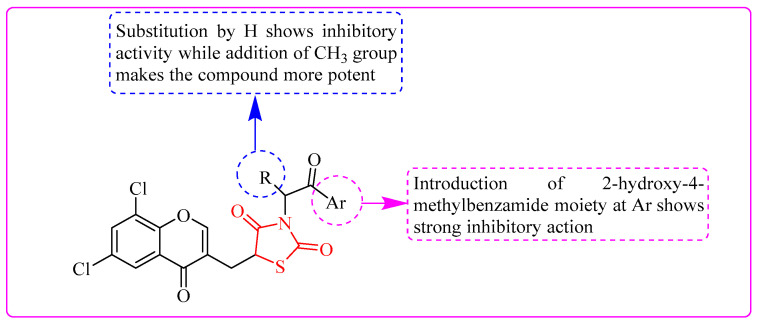
SAR for 5-((6,8-dichloro-4-oxo-4H-chromen-3-yl)methyl)-3-(1-substituted-2-oxo-2-arylethyl)thiazolidine-2,4-dione.

According to the literature, 6,8-dichloro-4-oxo-4H-chromene-3-carbaldehyde **142** refluxed for 3 h with thiazolidinedione **1** and anhydrous sodium acetate in acetic acid, further produced the final compound [5-(6,8-dichloro-4-oxo-4H-chromen-3-ylmethyle55ne)-2,4-dioxo-thiazolidin-3-yl]-acetaldehyde **144** with the help of dimethylformamide (DMF) in KOH, as outlined in Scheme 28. All the derivatives were tested for their antimicrobial activity against Staphylococcus aureus ATCC 49444, Listeria monocytogenes ATCC 13932, Salmonella typhimurium ATCC 14028, Candida albicans ATCC 10231, and Escherichia coli ATCC 25922. The concentrations of 1 mg/mL, 5 mg/mL, and 10 mg/mL of compounds were used and these results were evaluated by the measurement of the inhibition zone diameters compared to gentamicin and fluconazole, respectively, as reference drugs. In this study compound, 4-{2-[5-(6, 8-Dichloro-4-oxo-4H-chromen-3-ylmethylene)—2,4-dioxo-thiazolidin-3-yl]-acetyl}-2-hydroxy-benzamide was found to be more active. The SAR analysis in Figure 29 shows that hydrogen substitution retains inhibitory activity, while methyl substitution enhances activity [75].

**Scheme 29:**
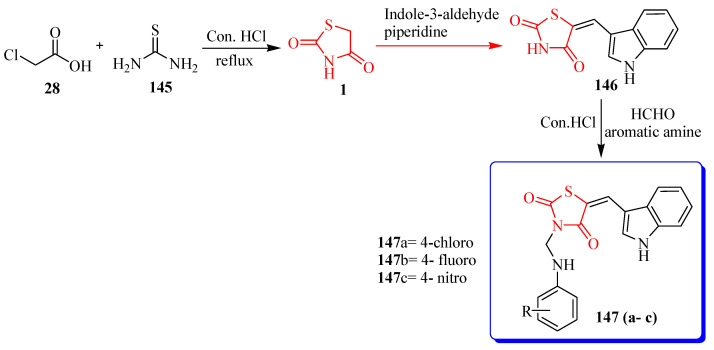
Synthesis of thiazolidinedione derivatives **147(a–c)**.

**Figure 30:**
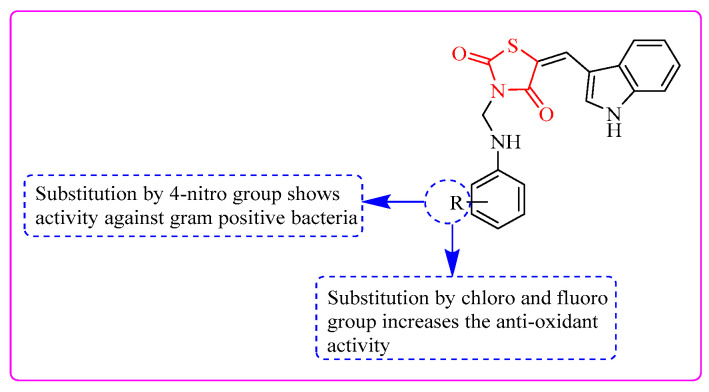
SAR of thiazolidinedione derivatives **147(a–c)**.

Initially, thiazolidine-2,4-dione **1** was synthesized conventionally according to the literature procedure [40,41]. Thiazolidine-2,4-dione **1** was condensed with indole3-aldehyde to form 5-[(indol-3-yl)methylene]-thiazolidine-2,4-dione **146** under Knoevenagel reaction conditions [42]. Compound **146** was then coupled with formaldehyde and substituted aromatic amines under Mannich reaction conditions [43,44] to afford the desired final derivatives **147(a–c) [76]**, as described in Scheme 29. Compound **147c** has demonstrated strong activity against *Gram-positive* bacteria (*B. subtilis* and *S. aureus*) at a concentration of 40 mg/mL, while compound **147e** has shown effective activity against *Gram-negative* bacteria (*E. coli* and *P. aeruginosa*) at the same dosage. In vitro antioxidant tests indicated that compounds **147a** and **147b** exhibited significant antioxidant effects in both the DPPH assay and the hydrogen peroxide assay methods. Evaluation of in vivo hypoglycemic activity showed that compounds **147a** and **147d** displayed promising hypoglycemic effects in both acute and chronic studies. Molecular docking analysis indicated that compound **147a** had the highest binding affinity for the PPARg receptor protein. The SAR in Figure 30 explains that the substitution with the nitro group (EWG) enhances activity against Gram-positive bacteria, and fluoro and chloro (EDG) show good antioxidant activity.

### 2.4. Antioxidant Activity

**Scheme 30:**
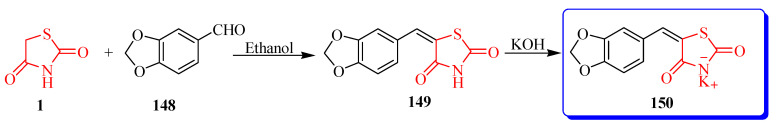
Synthesis of potassium salt derivatives of thiazolidinedione scaffold.

**Figure 31:**
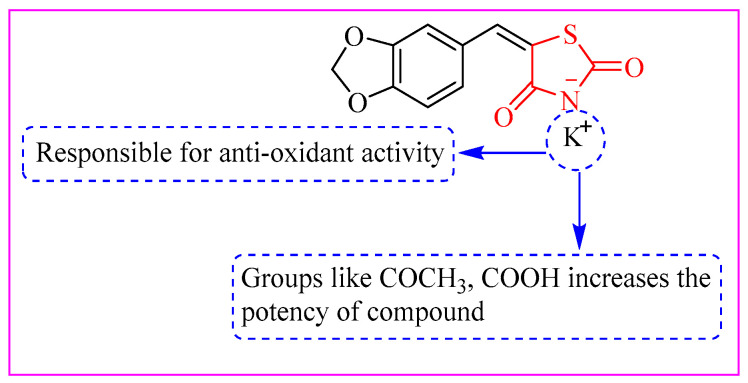
SAR of potassium salt derivatives of thiazolidinedione scaffold.

The 5-(benzo[d][1,3]dioxol-5-ylmethylene) thiazolidine-2,4-dione **149** reacted with potassium hydroxide in DMF to give the potassium salt derivative **150**, which is mentioned in Scheme 30 along with the SAR in Figure 31 [77].

**Scheme 31:**
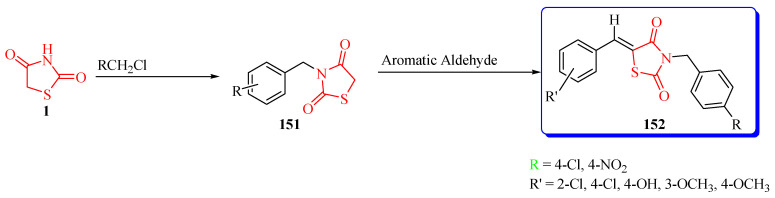
Synthesis of 5-aryl benzylidene-3-(4-substituted-benzyl)-thiazolidine-2,4-diones.

**Figure 32:**
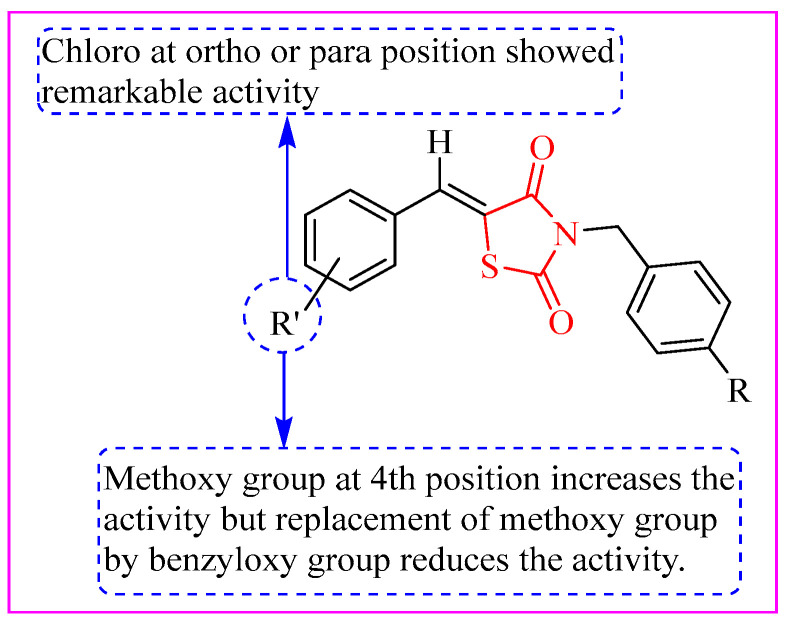
SAR of 5-Benzylidene-3-(4-methyl-benzyl)-thiazolidine-2,4-diones.

5-aryl benzylidene-3-(4-substituted-benzyl)-thiazolidine-2,4-diones **152** has been synthesized (Scheme 31) with the help of nucleophilic addition reaction starting from 2,4-thiazolidinedione **1** with benzyl or phenacyl halides in a hot alcoholic medium, which further leads to final product formation via the appropriate route of pioneer intermediate compound 3-(4-Methyl-benzyl)-thiazolidine-2,4-dione **151** synthesis. Among all the synthesized compounds, 5-(2,4-Dimethoxy-benzylidene)-3-(4-methyl-benzyl)-thiazolidine-2,4-dione compound was found to be most active. The SAR (Figure 32) of the synthesized compound illustrated that the “R” group on the benzene ring with a 4-nitro group enhances antibacterial activity, while chloro or fluoro substitutions increase antioxidant properties [78].

### 2.5. Anti-Inflammatory Activity

The literature indicates that the reactants 1-phenylethan-1-one **153** and phenylhydrazine **154** were mixed in ethanol and glacial acetic acid to produce (2E)-1-phenyl-2-(1-phenylethylamine)hydrazine **155**. The intermediate was subsequently treated to a Vilsmeier–Haack reaction to provide the important intermediate, substituted pyrazole carboxaldehyde **156**.

**Scheme 32:**
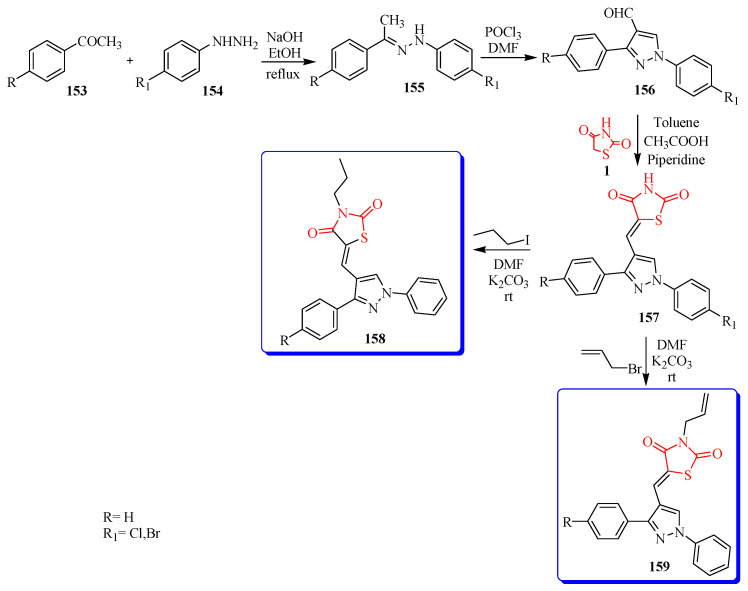
Schematic representation for synthesis of substituted thiazolidinediones and its derivatives.

**Figure 33:**
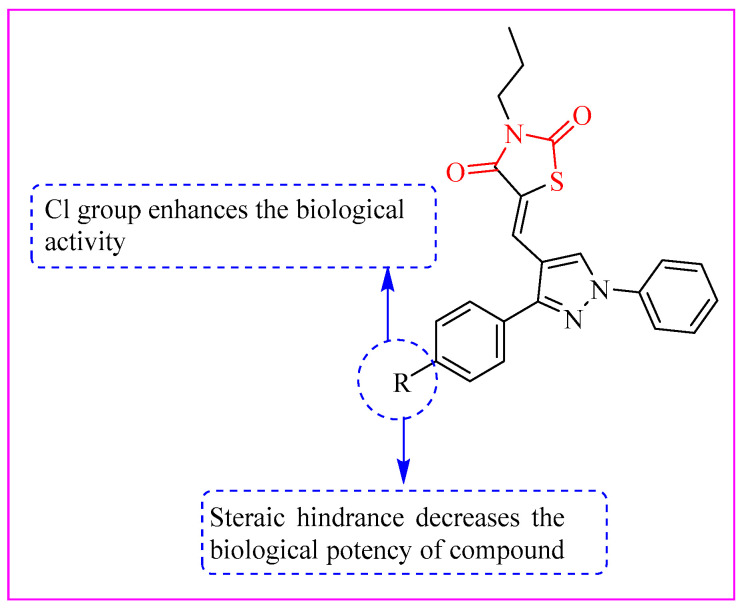
SAR of substituted thiazolidinediones and its derivatives.

Subsequently, 5-((1,3-substituted-1H-pyrazol-4-yl)methylene)thiazolidine-2,4-diones **157** was synthesized through the condensation of compound **156** with 2,4-thiazolidinedione **1** using piperidine and glacial acetic acid in anhydrous toluene. Compound **157** followed an additional reaction with allyl bromide and iodopropane in the presence of potassium carbonate in anhydrous DMF, yielding two products: 5-((3-substituted-1-phenyl-1H-pyrazol-4-yl)methylene)-3-propylthiazolidine-2,4-diones **158** and 3-allyl-5-((3-substituted-1-phenyl-1H-pyrazol-4-yl)methylene)thiazolidine-2,4-diones **159** (Scheme 32). Among all produced compounds were 5-((3-(4-chlorophenyl)-1-(4-nitrophenyl)-1H-pyrazol-4-yl)methylene)thiazolidine-2,4-dione and 3-allyl. -5-((3-(4-chlorophenyl)-1-phenyl-1H-pyrazol-4-yl)methylene)thiazolidine-2,4-dione has been identified as an effective inhibitor of COX-1 and COX-2. SAR indicates that the steric hindrance can negatively affect activity by inhibiting the ability to bind effectively to its target (Figure 33) [79,80].

### 2.6. Miscellaneous Activity

**Scheme 33:**
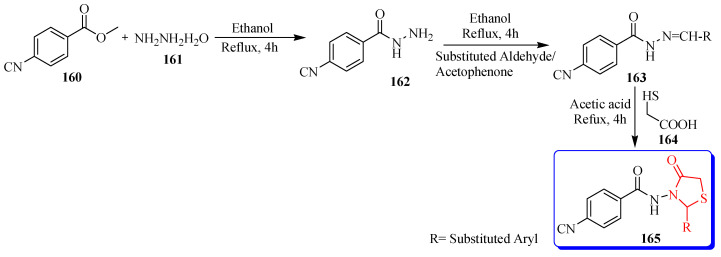
Synthesis of arylhydrazide-bearing thiazolidinone.

**Figure 34:**
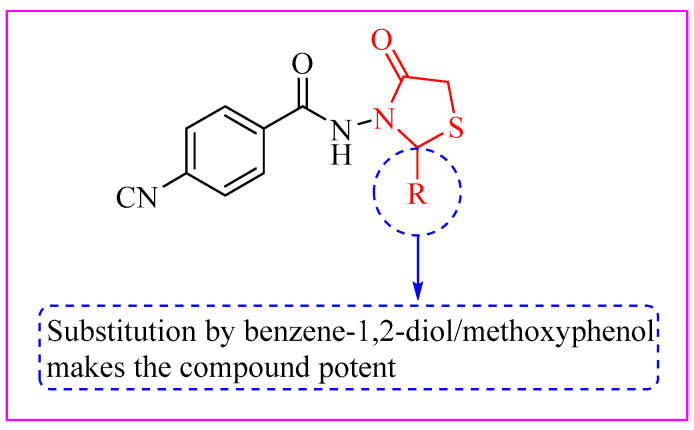
SAR of arylhydrazide-bearing thiazolidinediones.

Rahim et al. reported a new and effective approach for synthesizing aryl hydrazide-bearing thiazolidinediones and assessed their effectiveness as α-amylase and urease inhibitors (Scheme 33). In this protocol, 4-cyanobenzoate **160** and hydrazine hydrate **161** were refluxed with substituted aldehydes or acetophenones, resulting in the formation of the corresponding Schiff base **163**. The intermediate was subsequently reacted with thioglycolic acid **164** in the presence of acetic acid to yield the final product, thiazolidinedione **165**. Compounds with R groups like 3-methoxyphenol, 1,3-dichlorophenyl showed strong α-amylase inhibition (IC_50_: 0.8–1.3 μM). Other derivatives such as nitrophenyl and hydroxyphenyl showed moderate activity with higher IC_50_ values (4.1–19.8 μM). The detailed SAR is given in Figure 34 [81].

## 3. Patents Landscape (2015–2025)

Thiazolidinediones-containing compounds improve insulin sensitivity by offering key innovations in the molecular framework, providing promising drug design, discovery, and development. In this review, the synthesis of thiazolidinediones with diverse pharmacological profiles is explored, with a focus on patents filed between 2015 and 2025, as discussed in Table 1.

## 4. Clinical Trials and FDA Approved Drugs

Numerous methods were described in this review for the synthesis of thiazolidinedione and derivatives, and researchers continue to do their studies to determine pharmacological profiles along with their measuring safety and ensuring fewer side effects. Hence, employing the thiazolidinediones moiety in clinical trials. The clinical trial data of the compounds and FDA approved drugs of have been reported in Table 2 and Table 3.

## 5. Conclusions

2, 4-thiazolidinedione has a versatile pharmacological profile, such as being antidiabetic, anti-inflammatory, anticancer, antimicrobial, etc. The review focuses on the synthesis of thiazolidinedione and its derivatives with their immense action on PPAR-γ activation via agonistic effects, which makes them important for their pharmacological actions. Different derivatives with their structure–activity relationship created interest in researchers for further research on different analogs of thiazolidinedione with high potency and low toxicity, which further helps in the new drug discovery process and research. The structural flexibility of the thiazolidinedione core permits significant modifications, resulting in a variety of derivatives with various biological activities. Comprehensive structure–activity relationship (SAR) analyses have yielded significant understanding of the impact of various substitutions on the pharmacological response, potency, and selectivity of these compounds. These findings have not only enhanced the understanding of their mechanism of action but have also generated significant interest among medicinal chemists in for the design and synthesis of novel analogs with improved efficacy and reduce toxicity. The continuing structure–activity relationship-driven modifications and mechanistic studies of thiazolidinedione analogs are expected to significantly enhance future progress in drug discovery and development.

## 6. Future Directions

As per the bibliometric graph suggestion, there are several research processes underway for the search for an optimal anticancer agent using thiazolidinedione as the core structure. Apart from this, the review also suggests the search for a more potent antidiabetic drug with positively altered pharmacological parameters for human consumption. Hence, the research using the thiazolidinedione heterocyclic ring needs to be expanded for future results. Despite significant progress in the identification of thiazolidinedione (TZD) derivatives, there are still several challenges to overcome and chances to seize. Future studies should focus on creating more selective and less harmful TZD analogs, particularly to address well-known side effects like weight gain and cardiovascular risks which are associated with PPAR-γ agonism. New TZD-based scaffolds with multi-target capabilities can be found more quickly using computational methods like molecular docking and AI-aided drug design. Furthermore, there are promising avenues for examining TZDs’ therapeutic potential in conditions other than diabetes, such as cancer, neurological diseases, and inflammation. The clinical potential of TZDs can be expanded by additional study on targeted drug delivery, nanocarrier systems, and hybrid compounds that combine TZDs with other pharmacophores. Finally, to translate in vitro findings into real therapeutic applications, more in vivo and clinical research needs to be performed.

## Figures and Tables

**Table 1:** Overview of patents containing thiazolidinedione moiety from 2015 to 2025.

S. No.	Patent	Date of Patent	Patient Title	Description	Inventor
1.	EP4015000B1 (European Patent)	1 January 2025	Combination product containing limonin compound and thiazolidinedione [82]	This invention specifically relates combination products that comprise limonoid as well as thiazolidinedione compounds, used in the treatment/prevention of diseases like diabetes metabolic syndrome and associated ones.	Dong LI, Quan HAN, Liu HU, and LIan XUE
2.	US12083107B2 (US Patent)	10 September 2024	Pharmaceutical compositions comprising glitazones and nuclear factor-2 (Nrf2) activators [83]	The patent gave information about the pharmaceutical composition of PPAR agonist and Nrf2 activators along with the method for using them in combination in the treatment of arthritis, psoriasis, multiple sclerosis, inflammatory bowel disease (IBD), and asthma.	Bjoern Colin Kahrs
3.	CN116354901B (Chinese Patent)	26 April 2024	A thiazolidinedione compound and its preparation method and application [84]	The current invention discloses a thiazolidinedione compound with application and salts thereof, showing good LSD1 protein inhibitory activity in clinical research stage, and it is being further developed into antitumor drugs.	Sun Kai, Liu Yonggang, Zheng Yichao, and Zhao Bing
4.	US11931345B2 (US Patent)	19 March 2024	Thiazolidinedione analogs for the treatment of NAFLD and metabolic diseases [85]	Discloses thiazolidinedione analogs useful in the treatment of non-alcoholic steatohepatitis (NASH), non-alcoholic fatty liver disease (NAFLD), as well as other metabolic disorders.	Gerard R. Colca
5.	US11918569B2 (US Patent)	14 March 2024	5-deutero-2,4-thiazolidinedione derivatives and compositions comprising and methods of using the same [86]	This invention involves synthesis of deuterated derivatives, stereoisomers, and pharmaceutically acceptable salts with treatment methods thereof	Sheila DeWitt, Vincent Jacques, and Leonardus Van Der Ploeg
6.	US11918567B2 (US Patent)	5 March 2024	Deuterium-enriched pioglitazone [87]	In this patent, deuterium-enriched pioglitazone’s applications were described, which are pharmaceutically acceptable in the treatment of non-alcoholic steatohepatitis.	Anthony W. Czarnik
7.	WO2023244738A1 (WIPO)	21 December 2023	Thiazolidinediones for the treatment of muscular dystrophies [88]	Provides methods for the treatment of disorders such as muscle dystrophy, including Duchenne muscular dystrophy (DMD), limb-girdle muscular dystrophy (LGMD), and Becker muscular dystrophy (BMD), via administration of PPAR-gamma agonist.	Laszlo Nagy, Lee SWEENEY, Davud HAMMERS, and Andreas PATSALOS
8.	EP4003514B1 (European Patent)	4 October 2023	Crystalline forms of deuterium-enriched pioglitazone [89]	Pioglitazone, a USFDA approved drug, to improve glycemic control in type 2 diabetic patients. It is useful in treating metabolic disorder, PCOS, DM-2, non-alcoholic steatohepatitis, non-alcoholic fatty liver disease, and neurological disorder.	Laure Francoise Valerie NAVARRE, Emeline GARDETTE, Sebastien BOLZE, Sheila Dewitt, Vincent Jacques
9.	CN114702486B (Chinese Patent)	19 September 2023	Thiazolidinedione bridged metronidazole berberine compounds, and preparation method and application thereof [90]	The current invention discloses thiazolidinedione-bridged metronidazole and berberine compounds with preparatory methods thereof. The compound was found to be antibacterial against Gram-positive and Gram-negative bacteria, providing antimicrobial therapy and being more efficient.	Zhou Chenghe, Li Zhenzhen, Yu Mingyu, Sun Hang
10.	US11731963B2 (US Patent)	22 August 2023	Process for preparing 5-[[4-[2-[5-(1-hydroxyethyl)-2-pyridinyl]ethoxy]phenyl]-methyl]-2,4-thiazolidinedione and salts thereof [91]	In this patent, a process was disclosed for synthesis of compounds with thiazolidine moiety and the pharmaceutically acceptable salts thereof, with the process of synthesizing intermediates.	Ana Maria GARCIA COLLAZO, Wolter ten Hoeve, Johannes Nicholas Koek, Johannes B. M. Rewinkel, Sander DE WILDE
11.	CN115246869B (Chinese Patent)	28 July 2023	A kind of tripterine-thiazolidinedione derivative and its preparation method and application [92]	The current patent relates tripterine-thiazolidine derivatives, the preparatory method, and application thereof. The derivatives were tested in vitro for proliferation activity among test cells, A549, MCF7, HT29, LN229, HOS, Bel7402, and MRC-5, which showed significant antitumor activity, and some compounds showed better activity than tripterine. The tripterine-thiazolidine derivatives showed high efficacy against lung cancer.	Zhang Lei, Liu Yunhui, Fu Xuefeng, Wang Shaojie
12.	CN108685916B (Chinese Patent)	4 July 2023	Application of thiazolidinedione compounds in double-target treatment of pituitary growth hormone adenoma [93]	This invention relates to thiazolidinedione compounds in double-great treatment of pituitary growth hormone adenoma. Their salt, preparation, and inhibiting of cell expression growth hormone receptor (GHR). These compounds are effective in treating pituitary growth hormone adenomas.	Zhang Chaoyun, Zhao Yao, Zhang Yichao, He Min
13.	CA3037958C (Canadian Patent)	14 June 2022	Glitazones for topical application [94]	Glitazones like pioglitazones are discussed in this patent which treats dermatologic issues like keratosis pilaris (associated with a decreased number or sebocytes) as a topical treatment.	Jeffrey L. Sugarman
14.	US10787425B2 (US Patent)	29 September 2020	Thiazolidinedione derivative and use thereof [95]	In this patent, novel compounds were represented with use and pharmaceutical compositions thereof.	Hooh Cho, Eunjeong YOON
15.	JP6453224B2 (Japanese Patent)	5 November 2019	Combination of an anticancer agent, such as a tyrosine kinase inhibitor, and a STAT5 antagonist, preferably thiazolidinedione, to eliminate blood cancer stem cells in vivo to prevent the recurrence of blood cancer [96]	This patent provided the combination method in the prevention of the recurrence of leukemia, characterized by expression of BCR-ABL fusion protein that comprise: tyrosine kinase inhibitor inhibiting phosphorylation of BCR-ABL treating cancer and a peroxisome proliferator-activated receptor gamma agonist inhibiting signal transduction/transcription activator 5 (STAT5).	Stefan Prost, Marek Kirszenbaum, Dantek, Michael Le, Philippe Rousselot, Philippe Lebrouche
16.	ES2610623T3 (Spanish Patent)	28 April 2017	New synthesis of thiazolidinedione compounds [97]	The current patent provided an intermediate compound in synthesizing PPAR modulating compounds like thiazolidinedione in the treatment or prevention of metabolic disorders such as obesity, hypertension, inflammatory diseases, and diabetes.	James R. Zeller, Steven P. Tanis, Scott D. Larsen, Timothy Parker
17.	CN103180304B (Chinese Patent)	15 February 2017	Synthesis for thiazolidinedione compounds [98]	This invention gives novel methods to synthesize PPAR-gamma sparing compounds, like thiazolidinediones, useful in preventing/treating metabolic disorders, hypertension, obesity, diabetes, or inflammatory diseases.	S. P. Tanis, T. Parker, R. C. Gardwood, G. D. Atman III, J. R. Zeller
18.	CN104230915B (Chinese Patent)	17 August 2016	Phenylpiperazine derivatives containing thiazolidinedione and preparation method thereof and purposes [99]	The patent relates to a phenylpiperazine derivative bearing thiazolidinedione with preparation method. The moiety itself showed an inhibitory effect on cancer cell lines, hence it is used in the preparation of anticancer drugs.	Zhu Hailiang, Sun Juan, Wang Xinyi, Guo Fengjiao, Zhang Yang
19.	US9364465B2 (US Patent)	14 June 2016	Thiazolidinediones of omega-3 polyunsaturated acids as new insulin sensitizers for treating type 2 diabetes [100]	In this patent are thiazolidinedione derivatives of omega-3 fatty acids as insulin sensitizers, and use in the treatment of type 2 diabetes, CVD, renal anemia, Alzheimer’s disease, hypertriglyceridemia, obesity, metabolic diseases, and modulating activity of peroxisome proliferator-activated receptors (PPARs).	Om P Goel
20.	CA2677201C (Canadian Patent)	17 November 2015	Solid preparation comprising alogliptin and pioglitzone [101]	This patent described the solid preparation of pioglitazone, useful in diabetes, with superior dissolution properties and chemical and dissolution stability.	Kenji Nakamura, Kenichiro Kiyoshima, Junya Nomura

**Table 2:** Clinical trial data of some of the reported compounds.

S. No.	Title	Chemical Structure	Description	Status
1	Study of Pioglitazone in Sporadic Inclusion Body Myositis [102]	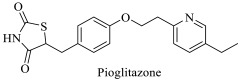	The clinical trial was performed to study the pioglitazone effects in skeletal muscle of sporadic inclusion body myositis (sIBM) patients.	Phase-I
2	Rosiglitazone in Treating Patients With Liposarcoma [103]	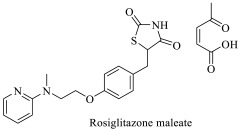	A phase II trial study, performed to estimate effectiveness of rosiglitazone in liposarcoma patients.	Phase-II
3	Pharmacokinetic and Biomarker Study of Pioglitazone in Adolescents With Severe Sepsis and Septic Shock [104]	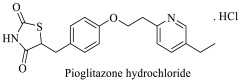	This study was performed to assess pharmacokinetics and effects on inflammatory biomarkers for pioglitazone in patient having severe sepsis and septic shock.	Phase-I & II
4	Study of Rivoglitazone in Type 2 Diabetes Mellitus [105]	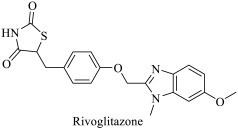	A 26-week study with type 2 diabetes patients, controlled by diet and exercise or with non-thiazolidinedione monotherapy.	Phase-III
5	Food Study of Pioglitazone HCL Tablets 45 mg to Actos Tablets 45 mg [106]	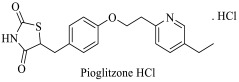	This trial assessed bioequivalence of Mylan pioglitazone HCl 45 tablets to Takeda Actos 45 mg tablets, followed by a single oral dose of 45 mg under fed conditions.	Phase-I
6	Pioglitazone Hydrochloride in Preventing Head and Neck cancer in Patients With Oral Leukoplakia [107]	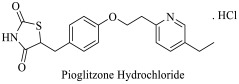	In this phase II trial, it was evaluated how pioglitazone hydrochloride prevents head and neck cancer in oral leukoplakia patients.	Phase-II
7	GlitazOne Treatment for Coronavirus HypoxiA, a Safety and Tolerability Open Label With Matching Cohort Pilot Study (GOTCHA) [108]	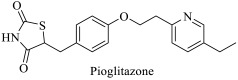	Pioglitazone is an approved anti-hyperglycemic medication which may have anti-inflammatory action. This trial was performed to study safety and tolerability related to the drug in elevated blood sugar level patients requiring hospital admission due to positive COVID-19 infection who did not received pioglitazone during hospitalization.	Phase-IV
8	Comparison of Lobeglitazone With PIoglitazone as Initial Triple Therapy for Diabetes Management [109]	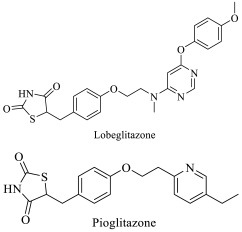	This trial has investigated the triple combination of metformin, DPP4 inhibitor, and thiazolidinedione in treating drug-naïve type 2 diabetes in Korean patients.	Phase-IV
9	Integrating the Genetic and Metabolic Faces of Obesity [110]	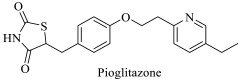	The study was performed to determine why obese subjects develop resistance to insulin. To evaluate obese subjects with characteristics of adipocytes and gene expression, and then entail them to either weight loss or treatment with pioglitazone, with the changes to be evaluated.	Phase-I
10	Randomized, Double-blind, Active-controlled, Study of Rivoglitazone in Type 2 Diabetes Mellitus [111]	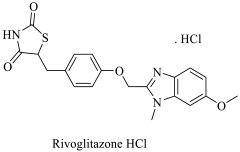	A randomized, double-blinded, 26-week study was performed in subjects who have type 2 diabetes controlled by diet and exercise or with non-thiazolidinedione monotherapy.	Phase-III
11	Evaluate the Drug-drugs Interaction Between Lobeglitazone 0.5 mg and Empagliflozin 25 mg [112]	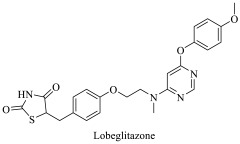	This clinical trial study was performed for the evaluation of pharmacokinetic parameters as well as safety of lobeglitazone in healthy individuals in a fasting state.	Phase-I
12	Pioglitazone Versus Empaglifozin of Chronic Pancreatitis/Recurrent Acute Pancreatitis Associated Diabetes Mellitus (PEP-DM) [113]	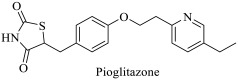	This clinical trial was performed to study the pioglitazone vs. empagliflozin efficacy in improving glycemic control with chronic pancreatitis or acute pancreatitis associated with diabetes mellitus subjects, as well as mixed meal response evaluation of both drugs.	Phase-II
13	Placebo and Active Controlled Study of Rivoglitazone in Type 2 Diabetes [114]	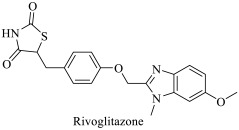	A 6-month study was performed to assess rivoglitazone’s monotherapy effects with use of placebo for comparison in newly identified type 2 diabetes/diabetes not adequately treated with other antidiabetic agents.	Phase-I & III
14	TriMaster: Study of a DPP4 Inhibitor, SGLT2 Inhibitor and Thiazolidinedione as Third Line Therapy in Patients With Type 2 Diabetes. (TriMaster) [115]	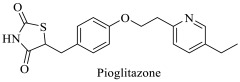	Identification of subgroups in type 2 diabetic patients who responds well/poorly to drugs based on clinical parameters like weight/kidney function, enabling a better target in treating the subjects.	Phase-IV
15	Comparison of Dapagliflozin, Lobeglitzone, and Its combination in Efficacy and Safety (Location-F) [116]	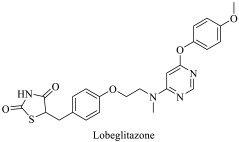	Reducing the visceral fat effect of combination therapy of DapaGliflozin (an SGLT-2 inhibitor) and lobeglitazone (a thiazolidinedione) was investigated to see which would be effective for diabetes treatment.	Phase II
16	Study to Evaluate the Safety, Tolerability and Efficacy of Three Dose Levels of Mitoglitazone in Type 2 Diabetic Patients [117]	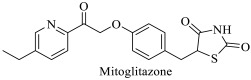	The trial has been completed for efficacy, safety, and tolerability of three dose level in patients with type 2 diabetes.	Phase II
17	A Clinical Study to Assess the Efficacy and Safety of Leriglitazone in Adult Male Subjects With Cerebral Adrenoleukodystrophy (CALYX) [118]	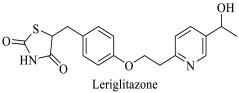	In the trial, efficacy and safety of leriglitazone were tested in adult male subjects with cerebral adrenoleukodystrophy.	Phase III
18	Ciglitazone [119]	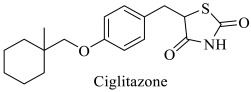	Developed in the 1980s by Takeda Pharmaceuticals, belongs to chemicals of phenol ethers, but discontinued in its in vitro studies.	Experimental

**Table 3:** FDA-approved drugs of class ‘glitazones’.

S. No	Name	Structure	Mechanism	Pharmacological Action	Year of Approval
1.	Pioglitazone	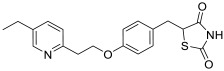	It activates PPAR-ƴ nuclear receptors. Hence, it increases insulin sensitivity [120].	Increases insulin sensitivity for the management of type 2 diabetes	1999
2.	Rosiglitazone	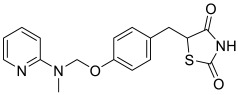	It activates PPAR-ƴ nuclear receptors. Hence, it increases insulin sensitivity [121].	Increases insulin sensitivity for the management of type 2 diabetes	1999
3.	Troglitazone	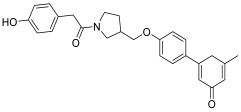	It activates PPAR-ƴ nuclear receptors. Hence, it increases insulin sensitivity [122].	Withdrawn (2000)	1997 (Approved)

## Data Availability

Not applicable.

## Abbreviations

PPAR-γ	Peroxisome proliferator-activated receptor gamma
TZDs	Thiazolidinediones
DMF	Dimethylformamide
PTSA	Para-toluenesulfonic acid
Cs_2_	Carbon disulfide
ANOVA	Analysis of Variance
SOCl_2_	Thionyl chloride
DCM	Dichloromethane
PEG	Polyethylene Glycol-600
POCl_3_	Phosphorus oxychloride
EtOH	Ethanol
Nrf2	Nuclear factor erythroid 2
IBD	Inflammatory bowel disease
NASH	Non-alcoholic steatohepatitis
NAFLD	Non-alcoholic fatty liver disease
DMD	Duchenne muscular dystrophy
LGMD	Limb-girdle muscular dystrophy
BMD	Becker muscular dystrophy
GHR	Growth hormone receptor
STAT5	Signal transduction/transcription activator 5
sIBM	sporadic inclusion body myositis
TNF	Tumor necrosis factor
IL	Interleukin
AMY2A	Amylase alpha 2A
GAA	Glucosidase alpha, acid
PPARG	Peroxisome proliferator-activated receptor gamma
PIK3CA	Phosphatidylinositol 4,5-biphosophate 3-kinase catalytic subunit alpha
PRKCB	Protein kinase C beta
INSR	Insulin Receptor
EDG	Electron donating group
EWG	Electron withdrawing group
CALYX	Cerebral adrenoleukodystrophy
SGLT-2	Sodium–glucose cotransport 2
DPP-4	Dipeptidyl peptidase-4
PEP-DM	Pharmacophore enhanced pharmacodynamic modeling
CVD	Cardiovascular disease
